# A new species of *Synagoga* (Crustacea, Thecostraca, Ascothoracida) parasitic in an antipatharian from Green Island, Taiwan, with notes on its morphology

**DOI:** 10.3897/zookeys.876.35443

**Published:** 2019-09-19

**Authors:** Gregory A. Kolbasov, Alexandra S. Petrunina, Ming-Jay Ho, Benny K.K. Chan

**Affiliations:** 1 White Sea Biological Station, Biological Faculty, Moscow State University, 119991, Moscow, Russia; 2 Invertebrate Zoology Department, Biological Faculty, Moscow State University, 119991, Moscow, Russia; 3 Biodiversity Research Center, Academia Sinica, Taipei 115, Taiwan

**Keywords:** Ascothoracida, black corals, lattice organs, live observations, morphology, parasitic crustaceans, SEM, taxonomy, ultrastructure

## Abstract

A new ascothoracidan species has been discovered off Taiwan in the north part of the west Pacific at SCUBA depths. Twelve specimens including both sexes of the new species, described herein as *Synagoga arabesque***sp. nov.**, were collected from colonies of the antipatharian Myriopathes cf. japonica Brook, 1889. Three previously described species of *Synagoga*, morphologically the least specialized ascothoracidan genus, have been found as ectoparasites of antipatharians and an alcyonarian, whereas all other records of this genus have been based on specimens collected from the marine plankton. This is the second study of a new form of *Synagoga* to be based on more than a few mature specimens of a single sex or on a single juvenile. Furthermore, it is the second in which SEM has been used to document the fine-scale external morphology. The position of terminal pores in the anterior pairs of the lattice organs is different in *Synagoga arabesque***sp. nov.** than those in *S. grygieri* Kolbasov & Newman, 2018 and *S. millipalus* Grygier & Ohtsuka, 1995. Species of *Synagoga* are small, host-specific predators or ectoparasites of antipatharians. This genus exhibits a major Tethyan reliction pattern.

## Introduction

Species of Ascothoracida are crustacean ecto-, meso-, and endoparasites of cnidarians (Scleractinia: Zoantharia, Antipatharia, and Alcyonacea) and echinoderms (Asteroidea, Crinoidea, Echinoidea, and Ophiuroidea). Currently this taxon is comprised of more than 100 described species assigned to two orders (Grygier1987a, 1996): Laurida, species of which are parasites of anthozoans except for those of *Waginella* Grygier, 1983a, which are ectoparasites of crinoids, and Dendrogastrida, species of which are parasites of non-crinoid echinoderms. Ascothoracidans are normally dioecious, the larger females being accompanied by smaller, sometimes dwarf, cypridiform males (Grygier and Fratt 1984; Grygier 1985a, 1987b, 1991a, b; Kolbasov 2007). However, members of Petrarcidae and possibly Ctenosculidae are simultaneous hermaphrodites (Okada 1938; Grygier 1983b, c). The life cycle of ascothoracidans includes up to six naupliar instars, one or two instars of a specialized ascothoracid larva, juveniles, and adults, but in a few species the naupliar phase is condensed or even omitted (Høeg et al. 2014). Depending on species, the larval stages may be free-swimming or brooded.

Members of the family Synagogidae represent the most generalized or basal group of ascothoracidans. Adult ascothoracidans belonging to such genera as *Synagoga* Norman, 1888, *Waginella* Grygier, 1983a, and *Sessilogoga* Grygier, 1990b are characterized by a bivalve carapace enclosing the whole body; the head bearing a pair of W-shaped, six-segmented prehensile antennules and an oral cone enclosing piercing mouthparts; the trunk consists of eleven segments, including six thoracomeres with biramous thoracopods, a genital somite bearing a (sexually dimorphic, vestigial in females) penis, three limbless abdominal somites, and the telson, with a pair of furcal rami.

Six described species and one unnamed ascothoracid larva are currently assigned to the genus *Synagoga*: the type species *S. mira* Norman, 1888 (see also Norman 1913) from the Bay of Naples; *S. normani* Grygier, 1983a from East Africa, *S. paucisetosa* Grygier, 1990a and *S. bisetosa* Grygier, 1990a (the latter only tentatively attributed to this genus) from the bathyal Atlantic, *S. millipalus* Grygier & Ohtsuka, 1995 from off Okinawa, *S. grygieri* Kolbasov & Newman, 2018 from the Azores and Cape Verde Islands, and “McKenzie’s larva” (Grygier 1988) from the eastern Indian Ocean (Table 2). Most of the descriptions were based on single individuals, and not always mature ones. Only *S. grygieri* was described on the basis of a number of specimens of both sexes (Kolbasov and Newman 2018), while *S. mira* was based on a few males. Furthermore, hosts have only been recorded for *S. mira* (the antipatharian *Parantipathes larix* (Esper 1788)), *S. normani* (an unidentified species of the alcyonarian *Dendronephthya* Kükenthal, 1905), and *S. grygieri* (the antipatharian *Antipathella wollastoni* (Gray 1857)).

The present study is the second, after that of *S. grygieri*, to describe a new species of *Synagoga* based on a number of specimens of both sexes with the extensive use of scanning electron microscopy (SEM) to document the fine-scale external morphology.

## Materials and methods

**Figure 1.**
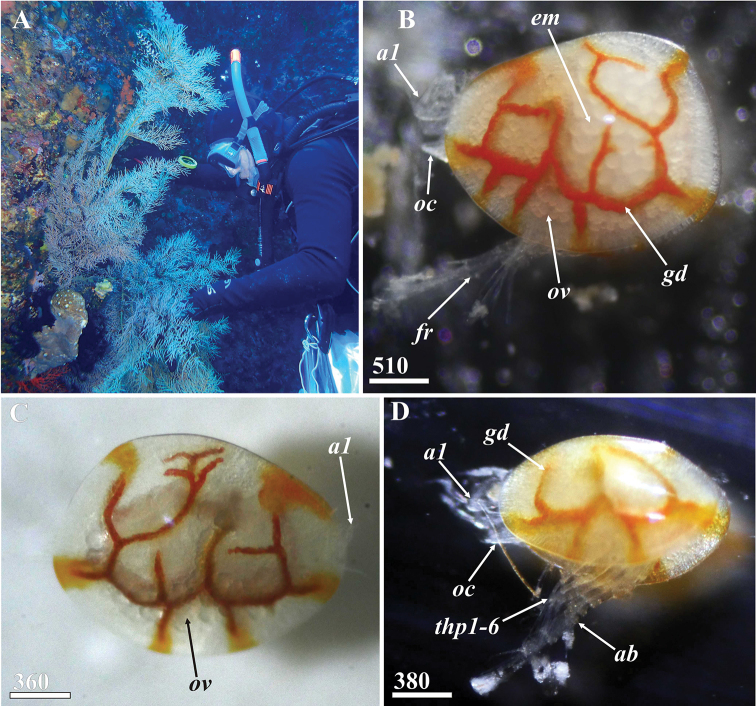
Collection and natural coloration of living specimens of *Synagoga arabesque* sp. nov. **A** Collection of living specimens of *Synagoga* from black coral *Myriopathes* sp. **B** mature female with outstretched antennules, oral cone and abdomen, lateral view, left side **C** young female, lateral view, right side **D** male with outstretched antennules, oral cone, thoracopods and abdomen, lateral view, left side. Abbreviations: *a1* – antennule, *ab* –abdomen, *em* – embryos, *fr* – furcal rami, *gd* – gut diverticulum, *oc* – oral cone, *ov* – ovary, *thp1-6* – thoracopods I-VI. Scale bars: in µm.

The ascothoracidans belonging to the new species *Synagoga arabesque* sp. nov. were collected alive from the two colonies of the antipatharian Myriopathes cf. japonica Brook, 1889. The colonies were first photographed and then collected alive in situ into sealed plastic bags (to prevent the escape of parasites) by GAK using SCUBA at depth of 35 m (Fig. 1A), at Green Island (Lyudao), Taiwan. Host specimens were transported in a portable ice box filled with seawater to the Green Island Marine Research Station, Biodiversity Research Center, Academia Sinica within 2 hrs of collection and subsequently maintained in an aquarium at 23–25 °C. Each colony was examined for crustacean parasites using stereomicroscope. The seawater from the sealed plastic bags was filtered through a sieve and the sample was also examined under the stereomicroscope. The ascothoracidans thereby discovered were fixed one-two days later in 100% alcohol, formalin, and glutaraldehyde, after digital photography using a Lumix (Panasonic) GH4 camera equipped with a Leica DG Macro-Elmarit 45 mm f2.8 lens and the same camera body affixed to an Olympus SZ61 dissecting microscope. Two females (holotype and paratype) and two males (paratypes) were dissected and mounted in glycerol on glass slides. They were examined and illustrated using a WILD Heerbrugg M20-35369 light microscope. Line drawings were also made using oil immersion, Nomarsky differential interference contrast, and a drawing tube on an Olympus BX 51 microscope. For SEM, three non-type females and two non-type males were post-fixed in 2% OsO4 for 2 h, dehydrated in acetone and critically-point dried with CO_2_. Dried specimens were sputter-coated with platinum-palladium and examined on a JEOL JSM-6380LA scanning electron microscope operating at voltages of 15–20 kV at the University of Moscow. Resulting photographs were touched up using CorelDraw X3 Graphics Suite.

## Systematics

### Subclass Ascothoracida Lacaze-Duthiers, 1880

#### Order Laurida Grygier, 1987a

##### Family Synagogidae Gruvel, 1905

###### Genus Synagoga

Taxon classificationAnimaliaLauridaSynagogidae

Norman, 1888

DE8BBC2B-493A-5359-BDDA-B124F7AEE4A3

####### Type species.

*Synagoga mira* Norman, 1888

###### Synagoga arabesque sp. nov.

Taxon classificationAnimaliaLauridaSynagogidae

73A9FAC5-DC97-55A3-8FC6-E8B3E7DAEBE5

http://zoobank.org/3BE6E08C-6AF5-45A9-946D-7DA1AF2BD63D

Figs 1 –18

####### Type locality.

Gongguan harbor, Green Island (Ludao), ca. 33 km off the southeastern coast of Taiwan, 22°41.438'N, 121°29.678'E, 35 m depth, 08 and 09 September 2017.

####### Material examined.

Twelve specimens of the new species, *Synagoga arabesque* sp. nov. (five males and seven females), were collected from two colonies of the antipatharian Myriopathes cf. japonica. Slides of the holotype female Mg 1243, and three paratypes (female, Mg 1244 and two males, Mg 1245) are deposited in the Zoological Museum of Moscow State University in Moscow, Russian Federation. The remaining two undissected paratypes (female and male) are deposited in alcohol in the Biodiversity Research Museum, Biodiversity Research Center, Academia Sinica, Taipei, Taiwan (ASIZCR000412). The other four SEM specimens and two undissected specimens in alcohol have been retained by the first author for further study and comparison with other synagogids.

####### Diagnosis.

Diagnoses for both adult females and males are provided for the new species, and a full list of interspecific differences is given in Table 2.

Females: carapace oval, slightly elongated in posterio-dorsal direction, up to 2.3 mm long and 2.0 mm high, with projecting posterio-dorsal tip. Massive setae (spines) of fourth antennular segment with row of dense, conspicuous denticles along anterior edge and rare, tiny denticles on posterior edge; fifth segment with 6–9 large setae; concave margin of antennular claw serrate in middle part. Exopod of second segment of thoracopod I with seven setae. Telson spines ca. 1/3 of blade length of furcal ramus; inner surface of furcal ramus with eight setae. Gut diverticulum red-orange, W-shaped, with numerous branches; dorsal, ventral, anterior and posterior branches terminate with light orange, wide areas at the edge of carapace.

Males: carapace ellipsoidal, up to 1.5 mm long and 0.9 mm high, with slightly projecting posterio-dorsal tip. Massive setae (spines) of fourth antennular segment differing slightly in length, with anterior and posterior rows of small denticles; fifth segment with 4–6 large setae; other characters of antennules similar to those in female. Exopod of second segment of thoracopod I with eight setae. Telson spines ca. 1/3 of blade length of furcal ramus; inner surface of furcal ramus with six setae. Gut diverticulum red-orange, W-shaped, with short anterior, posterior, and two ventral branches; branches terminate with light orange wide areas at edge of carapace.

####### Etymology.

From French *arabesque* borrowed from Italian *arabesco* - foliate ornament, used in the Islamic world, referring to the complex ornament of gut diverticula in carapace valves. The name *arabesque* has no appropriate equivalent in Latin and is used in this context as an arbitrary combination of letters (sensu ICZN Article 11.3) to avoid using the word in the vernacular.

####### Relation to host and behavior.

Animals were seen freely swimming from one branch of the antipatharian colony to another and represent small predators rather than ectoparasites. All live specimens of *Synagoga* were collected after washing the colonies. Animals were quite motile and moved in a Petri dish by jumping. To accomplish these jumping movements, they bent and unbent their developed abdomen with furca, while thoracopod beating was used for slow swimming.

####### Description.

**Figure 2.**
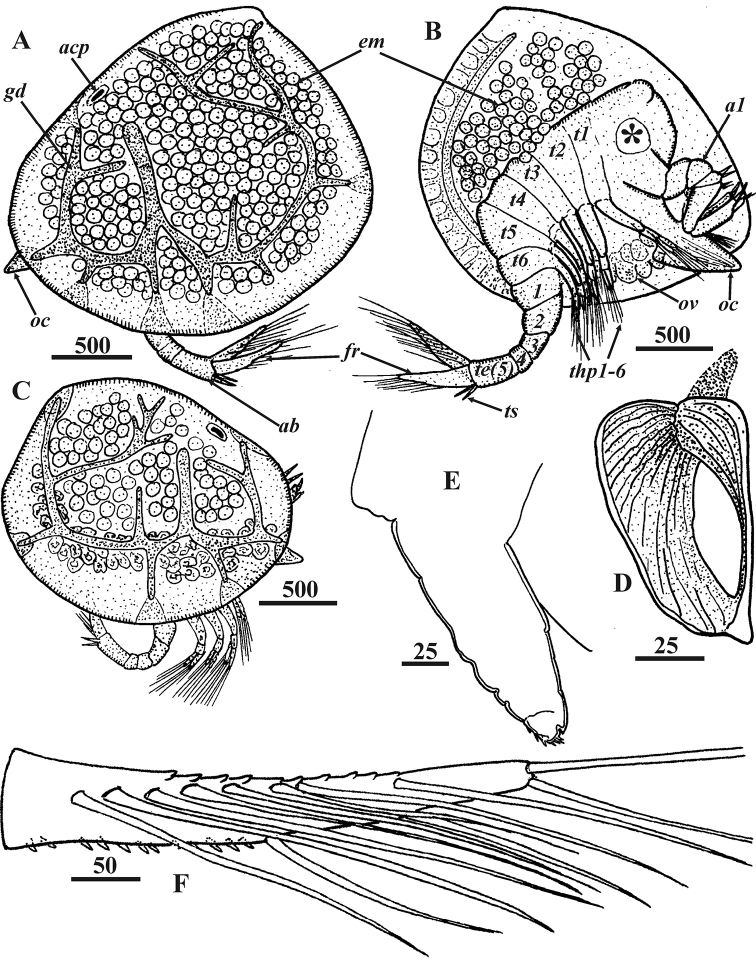
*Synagoga arabesque* sp. nov., female. General morphology **A, B, D–F** holotype **C** paratype Mg 1244 **A** general view lateral, left side **B** General view lateral, right valve of carapace removed, segments of thorax (*t1-6*) and abdomen (*1-5*), entrance of gut diverticulum and adductor muscle indicated by asterisk **C** General view lateral, right side **D** Anterior carapace pit, ventral end below **E** Rudimentary penis **F** furcal ramus, inner side. Abbreviations: *a1* – antennules, *ab* – abdomen, *acp* – anterior carapace pit, *em* – embryos, *fr* – furcal rami, *gd* – gut diverticulum, *oc* – oral cone (pyramid), *ov* – ovary, *t1-6* – segments of thorax, *te(5)* – fifth abdominal segment(telson), *thp1-6* – thoracopods I–VI, *ts* – telson spines. Scale bars: in μm.

Living specimens of both sexes semitransparent, light colored, but with bright red-orange gut diverticula; rounded embryos brooded inside female mantle cavity visible through carapace (Figs 1, 2). Abdomen and antennules often extending out of carapace during movements (Fig. 1B–D).

**Figure 3.**
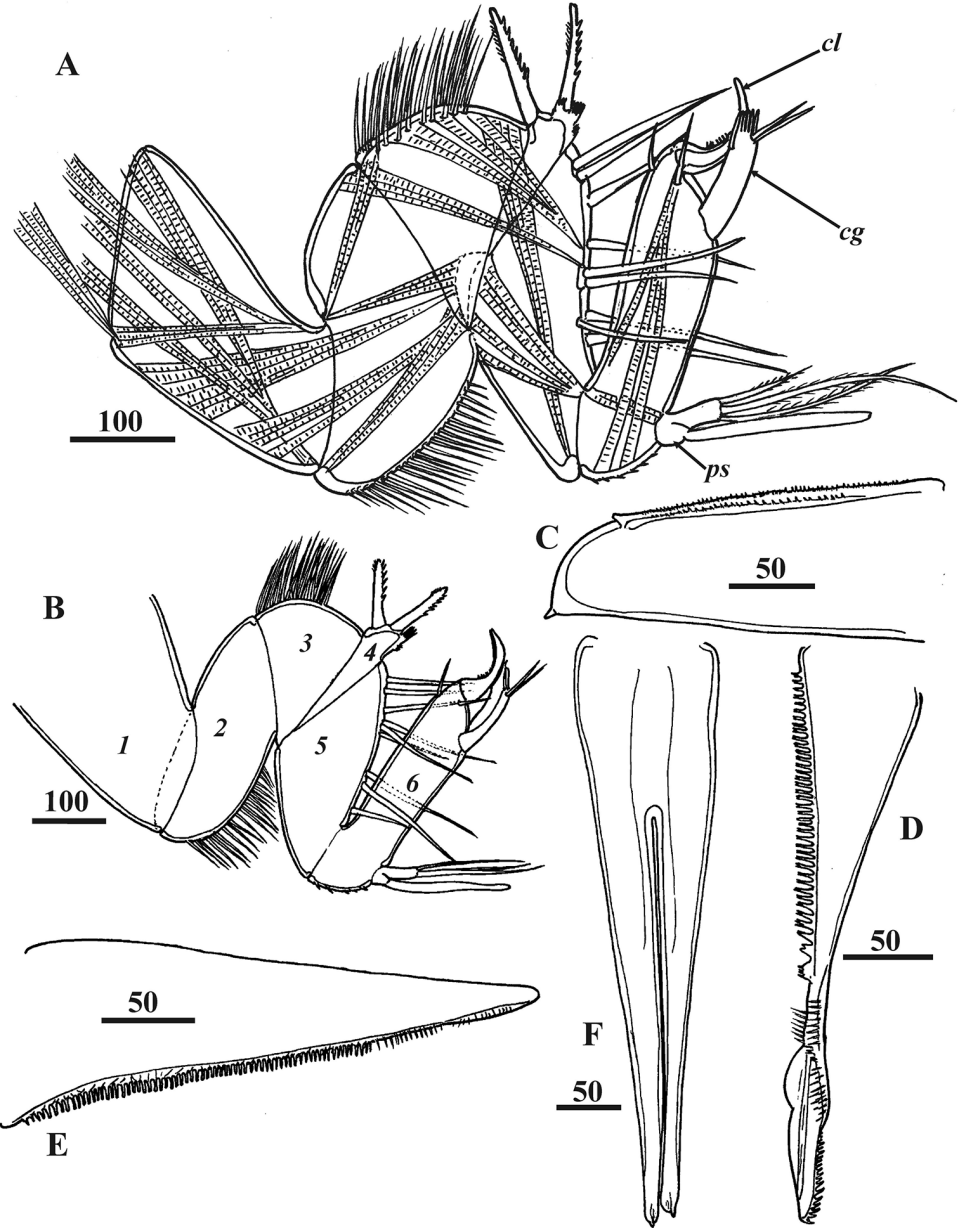
*Synagoga arabesque* sp. nov., female, holotype. Head appendages **A** right antennule with musculature **B** left antennules, segments numbered **C** medial languette **D** mandible **E** maxillule **F** maxillae. Abbreviations: *cg* – claw guard, *cl* – claw, *ps* – proximal sensory process. Scale bars: in μm.

**Figure 4.**
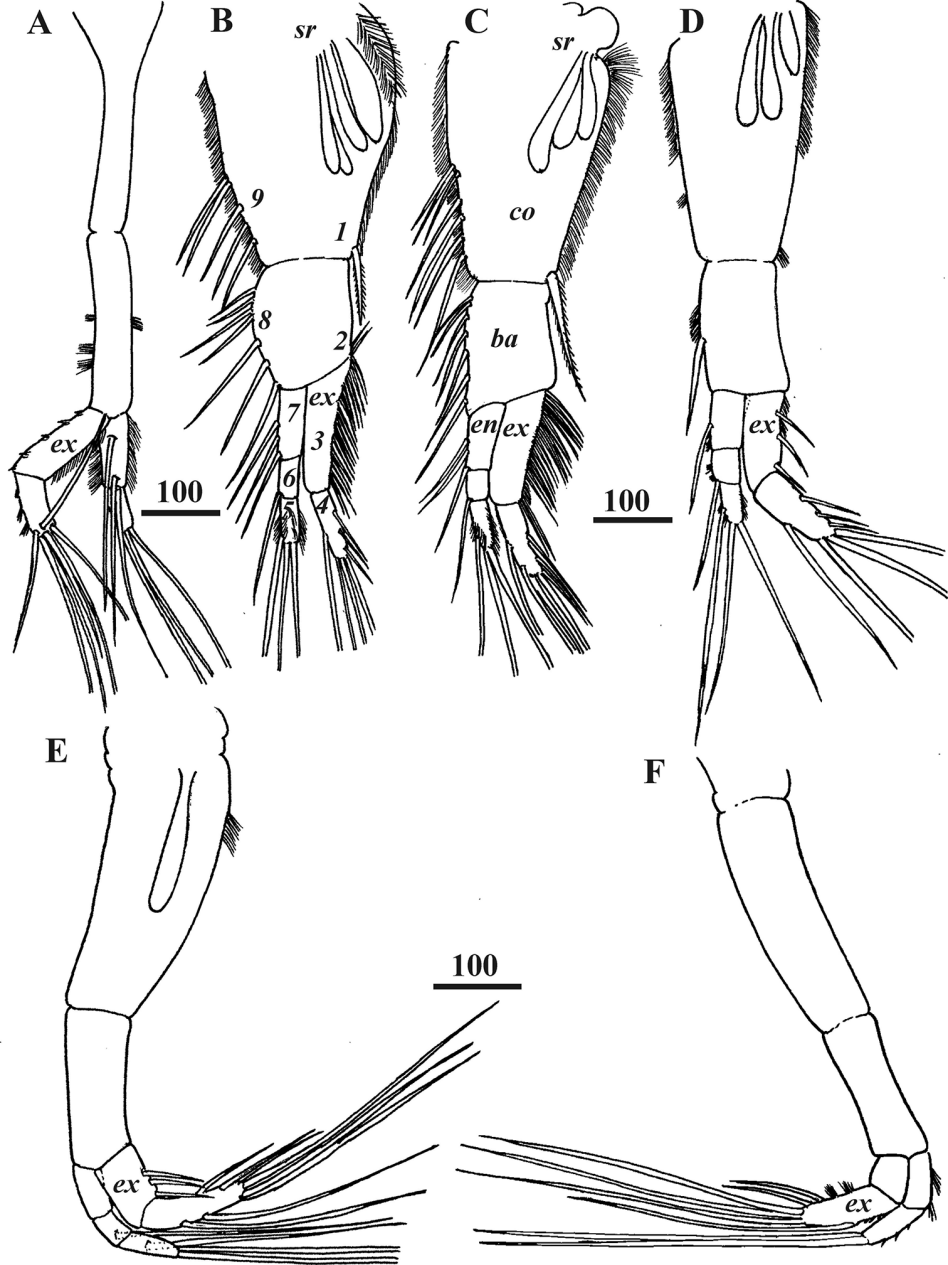
*Synagoga arabesque* sp. nov., female, holotype. Left (**A, B, D–F**) and right (**C**) thoracopods I–VI respectively. Ampuliform seminal receptacles are situated in upper outer parts of coxae of thoracopods II–V (**B–E**). Numbers indicating positions for setal counts in description (1–9) are shown for thoracopod II (**B**). Abbreviation: *ba* – basis, *co* – coxa, *en* – endopod, *ex* – exopod, *sr* – seminal receptacles. Scale bars: in μm.

Female (Figs 1–4, 8–12): Carapace oval, up to 2.3 mm long and 2.0 mm high, bivalved (Figs 1B, C, 2A–C, 8A, B), valves joined and hinged along dorsal margin (Fig. 17A). Dorsal and posterior margins of valves feebly convex, meeting at slightly produced posterio-dorsal angle; anterior and ventral margins rounded (Figs 1B, C, 2A–C, 8B).Exterior of carapace smooth, lacking setae but covered with small pores (Figs 8A, 17A, B, 18A–D). Right and left gut diverticula (Figs 1B, C, 2A–C) lying within respective carapace valve, resembling letter “W”; short main branch descending toward ventral margin and bifurcating, with anterior branch shorter than posterior and numerous simple and bifid small branches extending from them in various directions; dorsal, ventral, anterior and posterior small branches terminated with light orange, wide areas at edge of carapace (Fig. 1B, C). Inner surface of carapace valves with cuticular lining or mantle (Fig. 8B–F). Small, narrow pit on inner surface of anterior part of each valve (Figs 2A, C, 8E). Anterior pit of carapace infundibuliform, with wide entrance and long, narrowed internal part (Figs 2D, 8E); cuticle of pit wrinkled, with circular folds, small pores and volcano-shaped papillae (Fig. 8E, F). Body situated within mantle cavity (Figs 2B, 8A); oval brood chamber for embryos in posterior portion of each valve (Fig. 2B). Cuticular armament of mantle similar to that in *S. grygieri* (see Kolbasov and Newman 2018). Main cuticular structures of mantle arrayed along its margin: anterior and ventral sides with submarginal underlying folder consisting of dense row of cuticular projections forming fringe or palisade (Fig. 8C); anterior, ventro-posterior and posterior sides of mantle bearing long setae with short setules, these being absent ventro-anteriorly (Fig. 8B–D).

**Figure 5.**
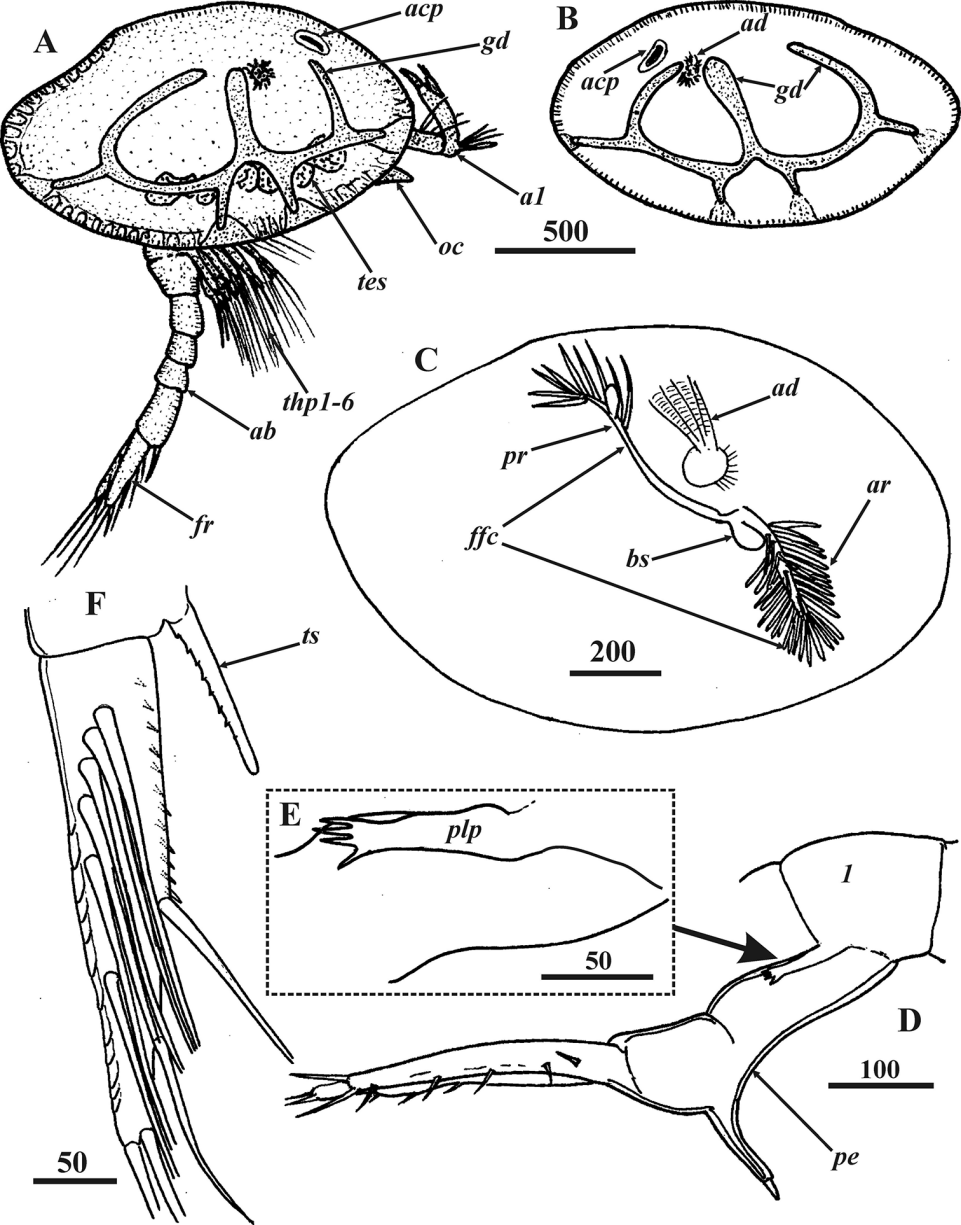
*Synagoga arabesque* sp. nov., male. General morphology **A, C–E** one paratype Mg 1245; **B** other paratype Mg 1245 **A** general view lateral with outstretched antennules, oral cone, thoracopods and abdomen, right side **B** general view lateral, left side, anterior end left **C** left valve of carapace with frontal filament complex, inner side, anterior end right **D** first abdominal (seventh trunk) segment with penis, lateral view **E** Enlarged part of basal shaft of penis with pleural process of first abdominal segment (*plp*) **F** Distal part of telson with telson spine and furcal ramus (inner side). Abbreviations: *a1* – antennule, *ab* – abdomen, *acp* – anterior pit of carapace, *ad* – adductor muscle, *ar* – anterior ramus of frontal filament complex, *bs* – basal ramus of frontal filament complex, *ffc* – frontal filament complex, *fr* – furcal rami, *gd* – gut diverticulum, *oc* – oral cone, *pe* – penis, *plp* – pleural process of first abdominal segment, *pr* – posterior ramus of frontal filament complex, *tes* – testis, *thp1-6* – thoracopods I–VI, *ts* – telson spine. Scale bars: in μm.

**Figure 6.**
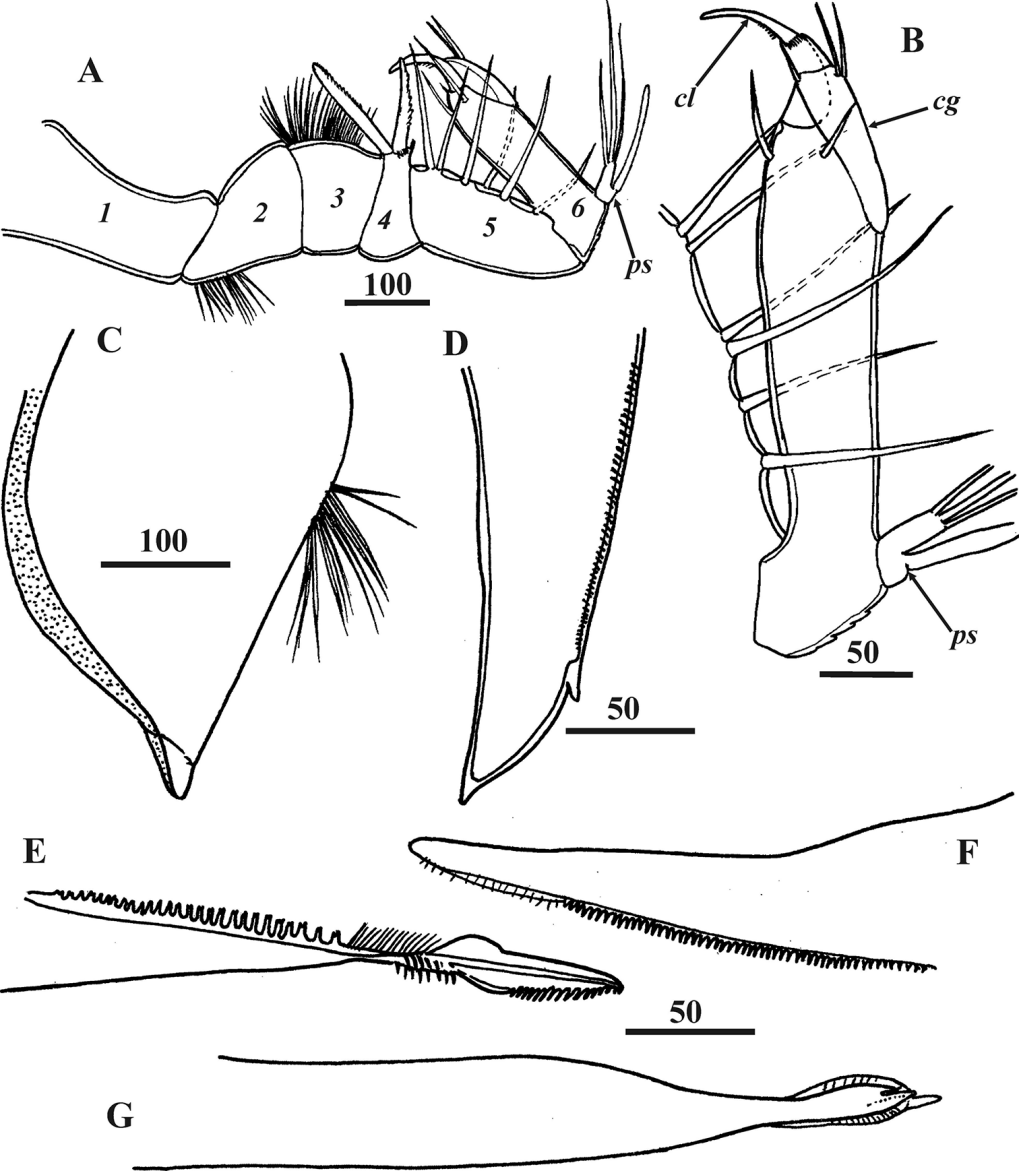
*Synagoga arabesque* sp. nov., male, paratype Mg 1245. Head appendages **A** left antennule, segments numbered **B** fifth and sixth antennular segments of right antennule **C** labrum lateral, anterior margin right **D** medial languette **E** mandible **F** maxillule **G** maxilla lateral. Abbreviations: *cg* – claw guard, *cl* – claw, *ps* – proximal sensory process. Scale bars: in μm.

Body proper consisting of unsegmented head and segmented thorax and abdomen. Head bearing W-shaped prehensile antennules followed by large ventral oral cone formed of mouth parts surrounded by labrum (Fig. 2B). Frontal filament complex (Fig. 8D) originating on mantle rather than body proper, ~380 μm long and trifid, with anterior ramus longest (ca. 350 μm) and densely covered by long, setiform protrusions; ampuliform, short basal ramus (ca. 70 μm) with smooth cuticle; and small, thin posterior ramus (ca. 40 μm).

Thorax consisting of six segments (Figs 2B, 9A, B), each with pair of biramous natatory thoracopods described in detail below. Dorsal sides of segments (II–VI) covered with thin setae (Fig. 9A, B). Posterio-ventral angles of sixth thoracic segment formed as small triangular projections or epaulets, their surface covered by rounded plaques (Fig. 9B).

Abdomen U-shaped, five-segmented, including telson (Figs 2B, 8A, 9B). First segment with vestigial penis on ventral side (Figs 2E, 9B, C), an unpaired process – 140–190 μm long, its distal part bearing ctenoid scales (Fig. 9C). Second segment trapezoid, bigger than either third or fourth. Last body segment (telson) cylindrical, ca. 300 μm long, its posterio-ventral margin bearing fringe of ctenoid scales and pair of conspicuous telson spines (Figs 2B, 9B, D) approximately 190 μm long with row of nine or ten sharp denticles along their dorsal margins. Furcal rami unsegmented (Figs 2B, F, 9B), approximately 410–560 μm long, thus approximately 2.5–2.9 times longer than telson spines; ventral margin with one medial, one subdistal and two distal setae, rarely with long setules (Figs 2F, 9B, E, G); proximal half of ventral margin armed with large, sharp denticles and ctenoid scales (Figs 2F, 9D, F, H). Inner subdorsal margin of each ramus with row of eight long natatory setae with long setules (Figs 2F, 9F, H); row of dense ctenoid scales along inner side of dorsal margin (Fig. 9F–H).

**Figure 7.**
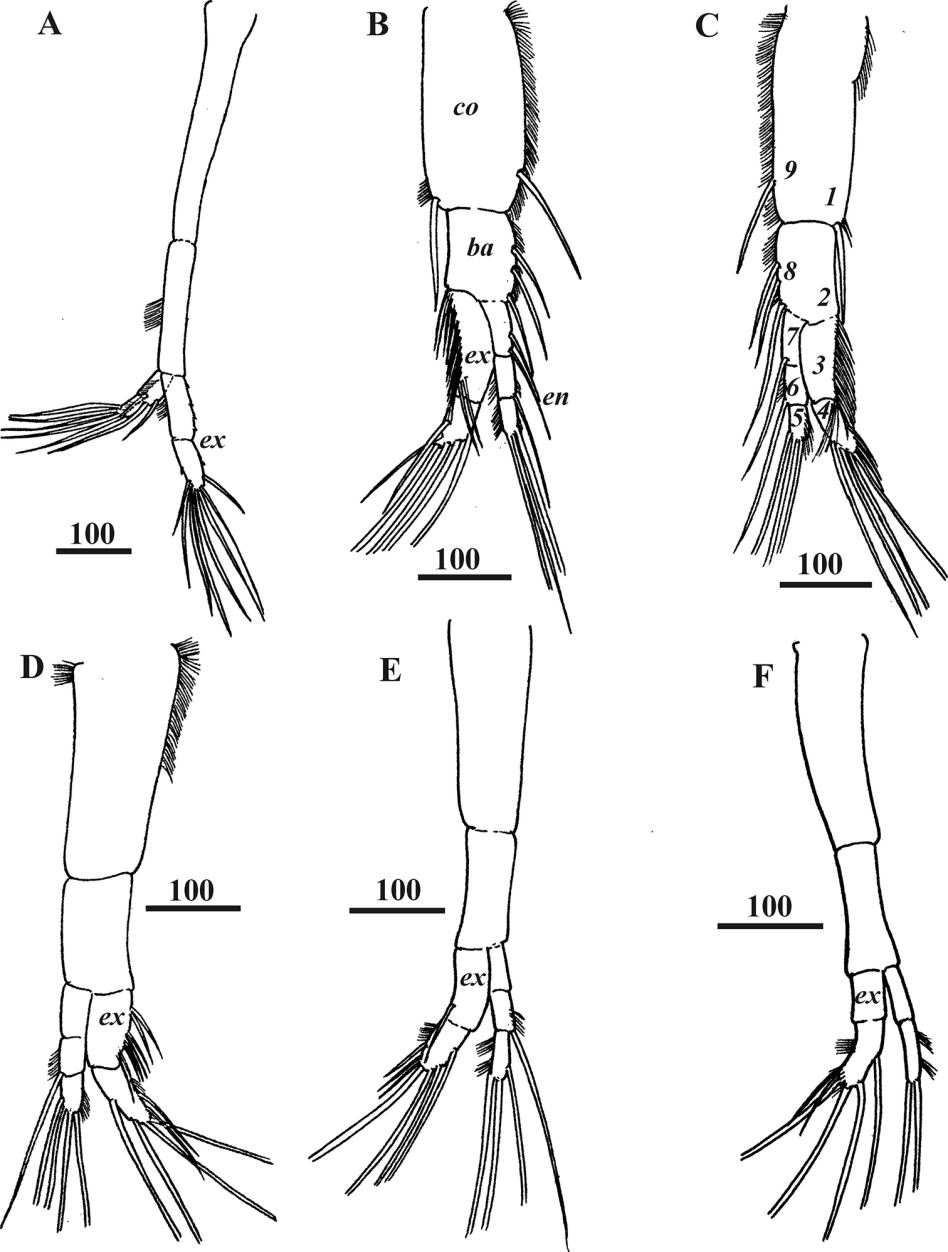
*Synagoga arabesque* sp. nov., male, paratype Mg 1245. Right thoracopods I–VI respectively (**A–F**). Numbers indicating positions for setal counts in description (1–9) are shown in thoracopod III (**C**). Abbreviations: *ba* – basis, *co* – coxa, *en* – endopod, *ex* – exopod. Scale bars: in μm.

**Figure 8.**
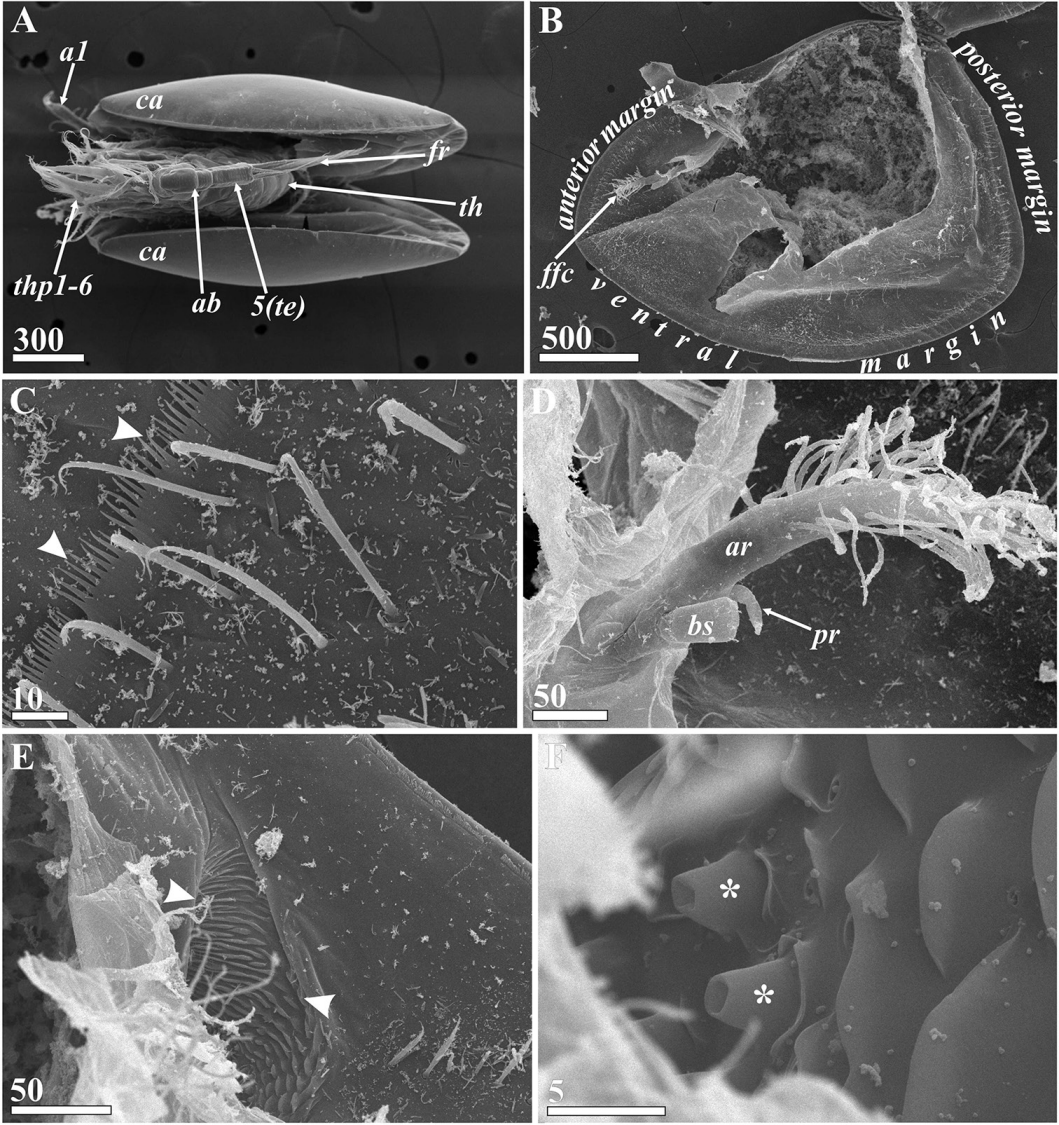
*Synagoga arabesque* sp. nov., female. General morphology, inner structures of carapace and mantle (SEM) **A** general view ventral **B** right valve of carapace, inner surface, mantle at place of body attachment (entrance of gut diverticulum and adductor muscle) destroyed **C** enlarged detail of mantle surface near anterior margin, submarginal fold of mantle with cuticular fringe (indicated by arrowheads) **D** frontal filament complex, anterior end left **E** Entrance of anterior pit of carapace (indicated by arrowheads) **F** surface of anterior pit of carapace (cuticular papillae indicated by asterisks). Abbreviations: *a1* – antennules, *ab* – abdomen, *ar* – anterior ramus of frontal filament complex, *bs* – basal ramus of frontal filament complex, *ca* – carapace (valve), *ffc* – frontal filament complex, *fr* – furcal ramus, *pr* – posterior ramus of frontal filament complex, *te* – telson, *th* – thorax, *thp1-6* – thoracopods I–VI. Scale bars: in μm.

**Figure 9.**
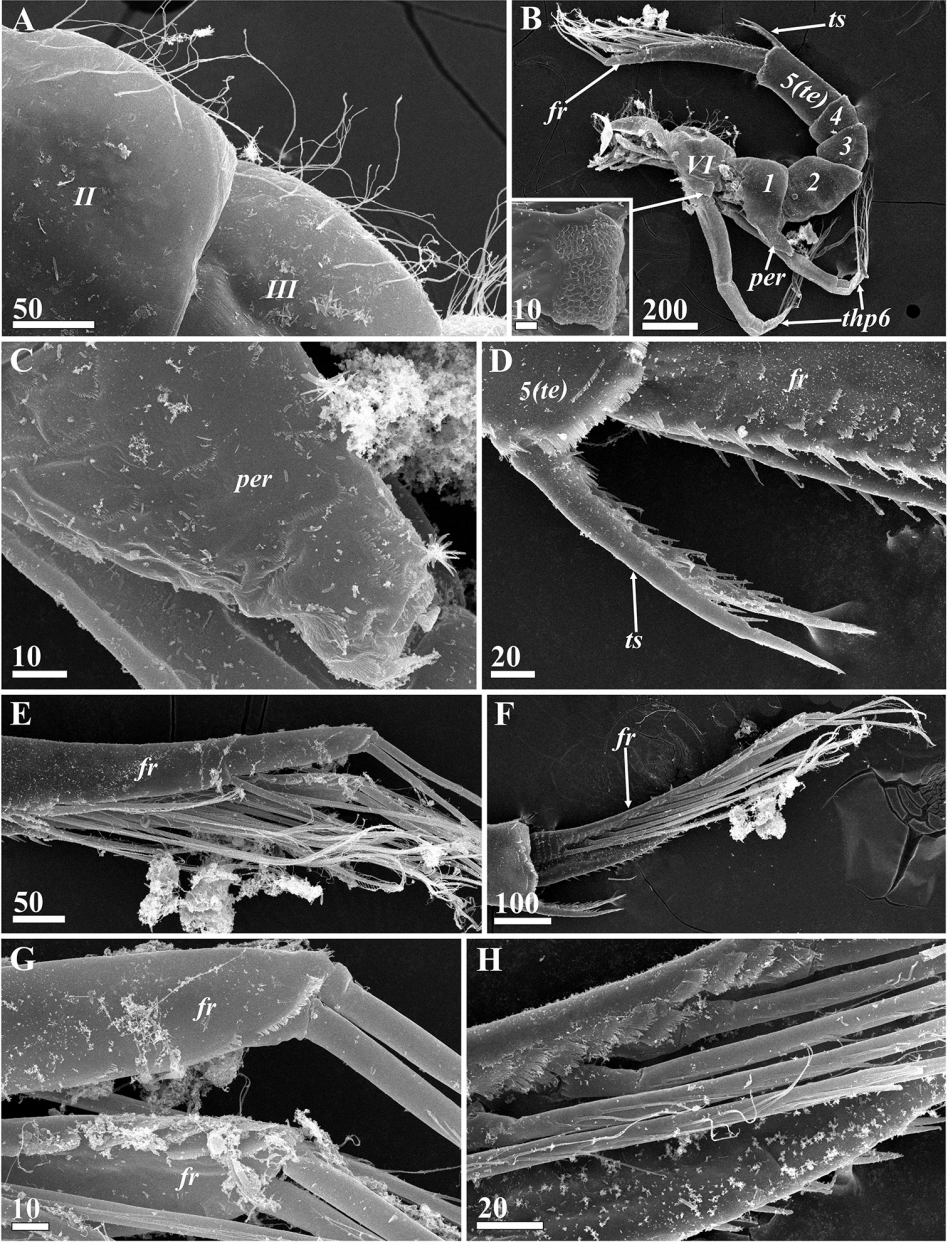
*Synagoga arabesque* sp. nov., female. Morphology and structures of thorax and abdomen (SEM) **A** dorsal surface of thoracic segments 2 and 3 **B** posterior part of thorax (segments numbered in Roman numerals) and abdomen (segments numbered in Arabic numerals), enlarged small epaulet in rectangle area in lower left angle **C** penis rudiment **D** telson spines **E** distal halves of furcal rami **F** furcal ramus, inner surface **G** terminal ends of furcal rami **H** Enlarged basal part of furcal ramus showing setation and sculpture on inner surface. Abbreviations: *fr* – furcal ramus, *per* – rudimentary penis, *te* – telson, *ts* – telson spines, *thp6* – thoracopod VI. Scale bars: in μm.

**Figure 10.**
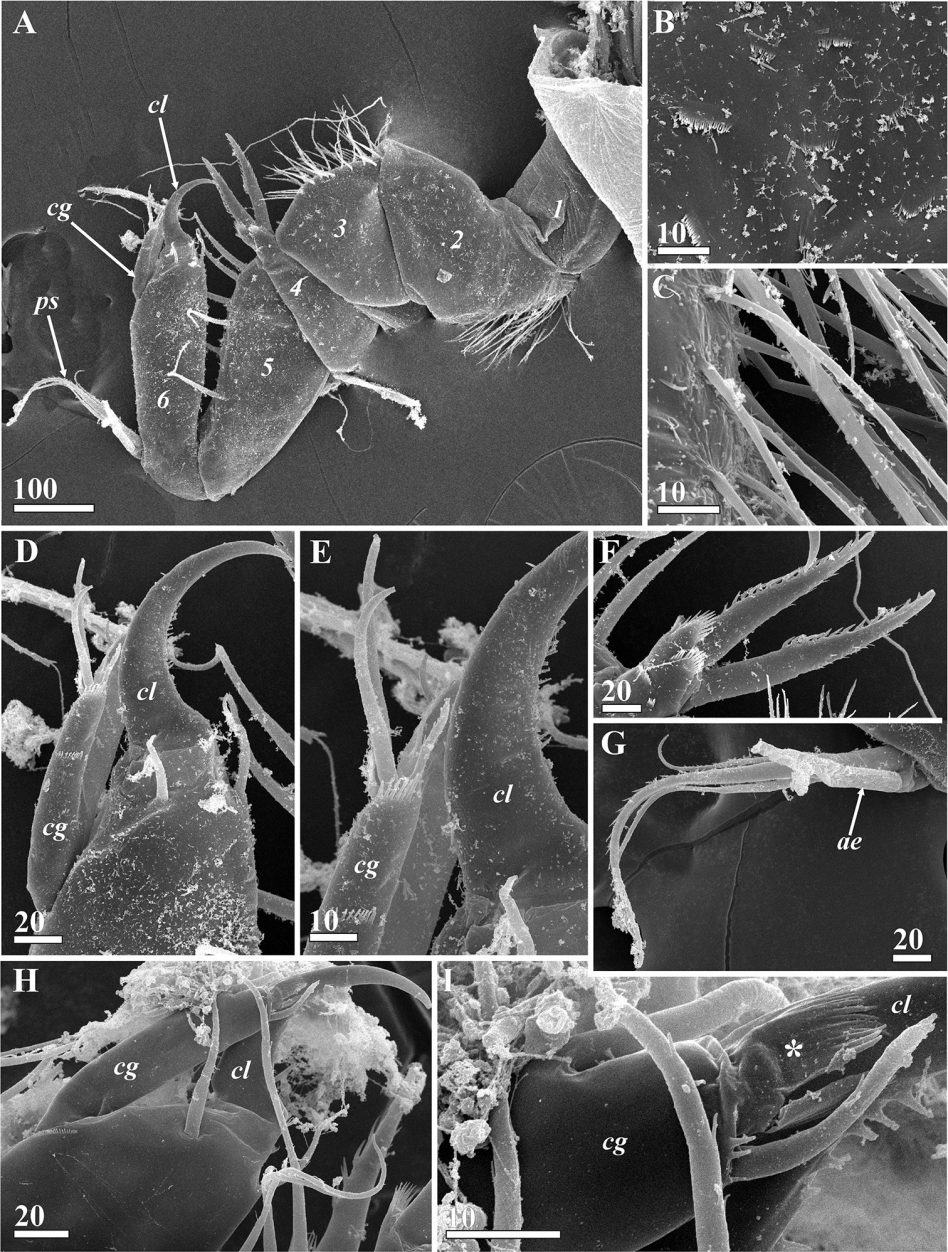
*Synagoga arabesque* sp. nov., female. Morphology of antennules (SEM) **A** right antennule, lateral view, inner surface, segments numbered **B** Ctenoid scales of second segment **C** omniserrate setae on postaxial/ventral surface of second segment **D** Claw sheathed by claw guard, inner side of sixth segment **E** Junction between claw and claw guard showing their microsculpture, inner side **F** Spines of fourth segment forming ‘fork’ to accept claw of sixth segment **G** proximal sensory process of sixth segment **H** claw sheathed by claw guard, outer side of sixth segment **I** junction between claw and claw guard showing their microsculpture, terminal ctenoid fold of claw guard sheathed claw indicated by asterisk, outer side. Abbreviations: *ae* – aesthetasc, *cg* – claw guard, *cl* – claw, *ps* – proximal sensory process. Scale bars: in μm.

Extendable, prehensile antennules subchelate, folded into W-shape, consisting of six segments with complex of intrinsic and extrinsic flexor and extensor muscles (Figs 2B, 3A, B, 10A). First segment rectangular, narrowing somewhat distally, without setae. Second segment irregularly rectangular, with dense, thin omniserrate setae along postaxial/ventral margin (Figs 3A, B, 10A, C). Third segment equilaterally triangular, narrowing toward ventral margin; preaxial/dorsal margin curved, densely covered by thin omniserrate setae (Figs 3A, B, 10A). Fourth segment rectangular, trapezoid (appearing triangular in folded antennules, Fig. 3A, B), very narrow, narrowing towards dorsal margin, with two massive and denticulate setae (‘spines’) armed with row of dense, conspicuous denticles along anterior edge and, rarely, tiny denticles on posterior edge; these two spines sitting on dorsal projection apex bearing ctenoid scales with sharp denticles (Figs 3A, B, 10A, F). The two spines form a fork to accept movable claw. Fifth segment conical, forming a palm against which sixth segment can fold in order to grasp host tissue, with 6–9 strong, simple setae along upper margin (Figs 3A, B, 10A). Sixth segment longer than fifth segment and armed with sensory and grasping structures (Figs 3A, B, 10A, D, E, G–I). Short proximal sensory process on lower margin at base of sixth segment (Figs 3A, B, 10A, G), with 3 terminal setae, middle one setulated and longest, and 1 thick, blunt sub-basal seta (at least this seta probably an aesthetasc). Curved claw on distal end of sixth segment apparently with muscles attached (Figs 3A, B, 10A, D, E); concave margin of claw serrate, with sharp tiny denticles in middle part (Fig. 10D, E); three small setae at base of claw, two lateral on inner and outer surfaces and one on anterior dorsal margin (Figs 3A, B, 10D, H). Relaxed claw sheathed by grooved claw guard (Figs 3A, B, 10D, E, H, I), latter approximately 110 μm long, with wide flange on inner side, thin, membranous, apical ctenoid hood (Fig. 10I) and four small terminal setae including two longer and one tiny subapical (Fig. 10E) and 1 tiny apical seta (Fig. 10I). Cuticle on sides of antennular segments bearing dense small ctenoid scales (Fig. 10B).

**Figure 11.**
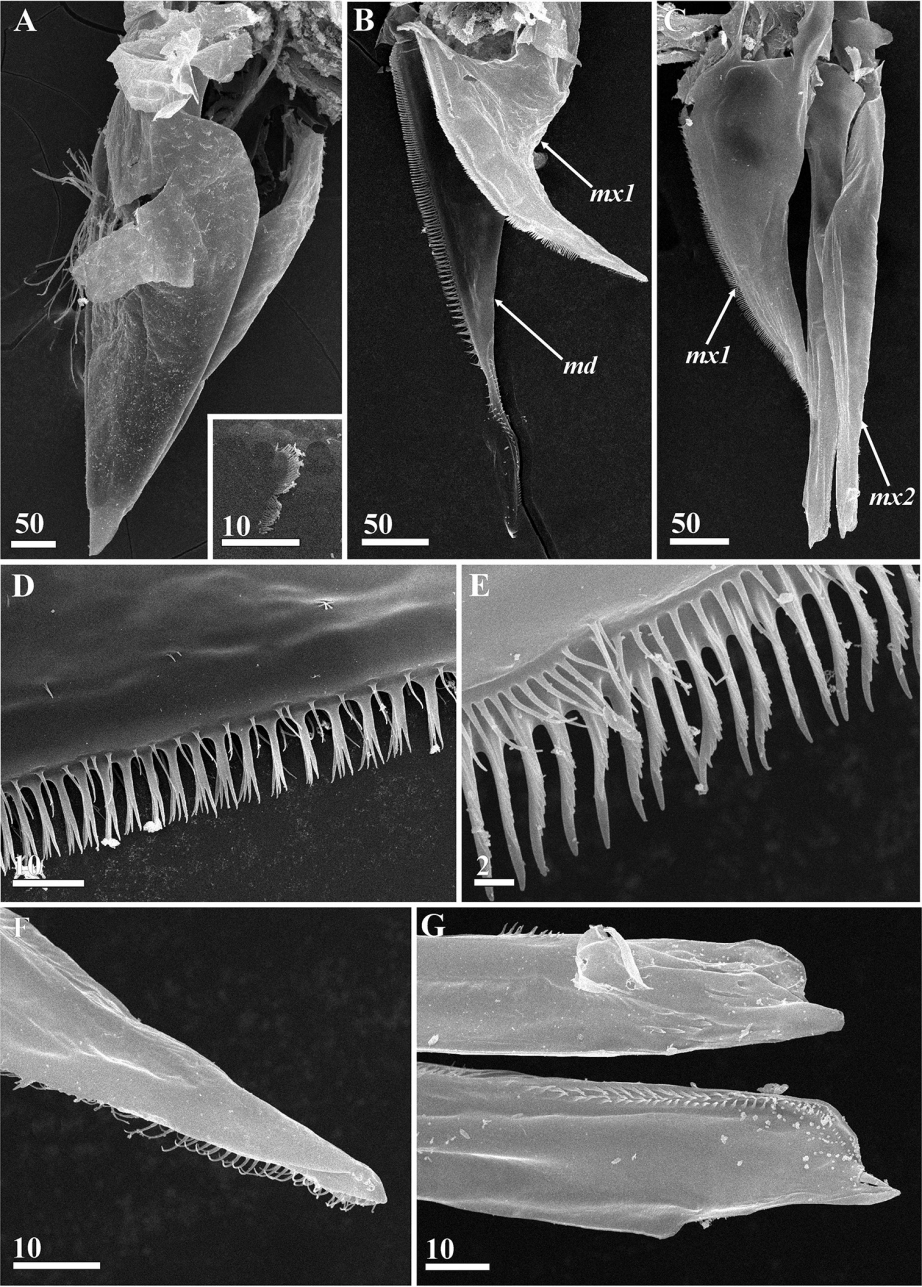
*Synagoga arabesque* sp. nov., female. Mouth parts (SEM) **A** labrum, posterio-lateral view, anterior margin left, enlarged ctenoid scales in rectangle area in lower right angle **B** mandible and maxillule (tip of mandible partially embedded in glue) **C** maxillule and maxillae **D** spines and setae along cutting (posterior) margin of mandible, middle part **E** spines and setiform projections along cutting (posterior) margin of maxillule, middle half **F** tip of maxillule **G** tips of maxillae. Abbreviations: *md* – mandible, *mx1* – maxillule, *mx2* – maxilla. Scale bars: in μm.

Oral cone prominent, approximately 600–650 μm long; distal end often protruding outside carapace (Figs 1B, D, 2A–C, 11A); formed by cone-shaped labrum surrounding piercing mouth parts (Fig. 11A). Posterior margins of labrum free, unfused (Fig. 11A). Tuft of long, thin simple setae in middle of anterior face of labrum; dense, small ctenoid scales on external cuticle (Fig. 11A). Mandibles in form of lanceolate stylets, approximately 350 μm long (Figs 3D, 11B); cutting edge of each bearing approximately 80–90 sharp, complex teeth with four tips (quadrifid), length of teeth increasing towards middle part of blade, with row of small setae paralleling them (Fig. 11D); neck of mandible lacking denticles or teeth but bearing small simple setae; distal part with row of 16–20 curved teeth on posterior margin (Figs 3D, 11B). Maxillules consisting of a wide basal half and narrow distal half (Figs 3E, 11B, C); cutting edge bearing numerous denticles with serrate margin and cuticular setiform projections, these denticles being massive in proximal part and thin and elongate in middle and distal parts (Fig. 11B, C, E); tip with thin, curved setiform projections (probably setae, Fig. 11F). Maxillae (Figs 3F, 11C) thin, fused at bases, with row of thin, needle-shaped denticles along inner cutting edge at distal end (Fig. 11G); tips not distinctly bifid, not harpoon-shaped, with apical projection and adjacent tiny process (probably seta, Fig. 11G). Unpaired process or medial languette (fused paragnaths?) originating from between paired mouth parts, with sharp tip and two rows of denticles on anterior margin (Fig. 3C).

**Figure 12.**
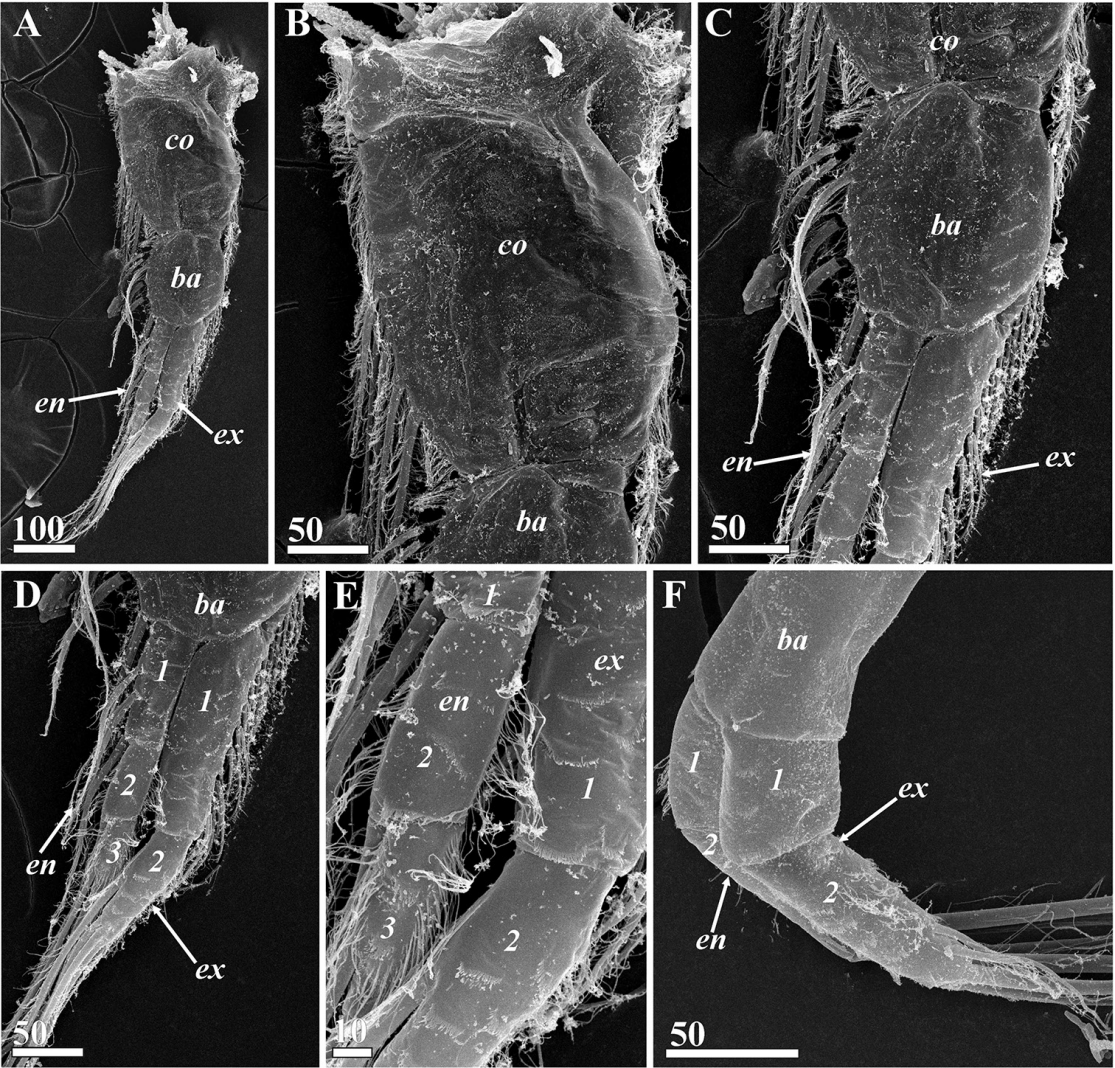
*Synagoga arabesque* sp. nov., female. Thoracopods (**A–E** – left thoracopod II, **F** – left thoracopod VI, SEM) **A** general view **B** surface and setation of coxa **C** surface and setation of basis **D** setation of rami, segments numbered **E** enlarged segments (numbered) of rami showing microsculpture **F** basis and rami, ramal segments numbered. Abbreviations: *ba* – basis, *co* – coxa, *en* – endopod, *ex* – exopod. Scale bars: in μm.

All thoracopods natatory and biramous (Figs 4, 12). Seminal receptacles found in lateral proximal parts of coxae of thoracopods II–V (Fig. 4B–E), consisting of ampuliform sacs with proximal parts converging but external opening(s) not observed; thoracopods II with four seminal receptacles, thoracopods III and IV each with three and thoracopod V with one. Thoracopodal setation summarized in Table 1. First thoracopod (Fig. 4A) slightly separated from others, with elongate protopod comprised of coxa and basis and two-segmented exopod and endopod; margins of basis with tufts of short setae; segments of exopod with ctenoid scales and small denticles, inner margin of basal segment lined with dense thin, small setae; seven long, plumose setae situated at distal end of second segment; basal segment of endopod bearing three long, plumose setae, margins being lined with dense thin, small setae; distal segment with three terminal plumose setae. Thoracopods II-V with three-segmented endopods and two-segmented exopods (Figs 4B–E, 12A–E). Coxae of thoracopods II and III (Figs 4B, C, 12B, C) with large, distal seta in position “1” (see Table 1 for further explanation) and row of plumose setae along inner edge (position “9”); these setae absent on coxae of thoracopods IV and V (Fig. 4D, E). Number of setae on rami of thoracopods II and III much more numerous than on thoracopods IV and V. Protopod of thoracopod VI (Figs 4F, 12F) narrow; coxa and basis without setae; both rami two-segmented with long, plumose terminal setae on distal segments; two tufts of thin, small setae on basal segment of endopod and distal segment of exopod. Surface of all thoracopods bearing conspicuous ctenoid scales (Fig. 12).

**Table 1.** Thoracopodal setation in *Synagoga arabesque* sp. nov. (ignoring tiny setae). Roman numerals indicate thoracopods I–VI. Positions 1–9 are indicated in Figs 4B and 7C. Question marks indicate that the position in question was obscured. Parentheses in thoracopods I and VI are used for the 2-segmented (instead of 3-segmented) endopods.

Position on thoracopods										
	1	2	3	4	5	6	7		8	9
♀										
I	0	0	0	7	3	(	3	)	0	0
II	1	0	30?	7	3	1	3		7	5
III	1	0	24?	7	3	1	3		7	7
IV	0	0	3	7	3	1	1		1	0
V	0	0	3	8	3	1	0		0	0
VI	0	0	0	6	2	(	0	)	0	0
♂										
I	0	0	0	8	4	(	3	)	0	0
II	1	0	16?	6	3	1	2		3	1
III	1	0	20?	7	3	1	1		3	1
IV	0	0	8	6	4	1	0		0	0
V	0	0	1	7	3	1	0		0	0
VI	0	0	0	6	2	(	0	)	0	0

**Figure 13.**
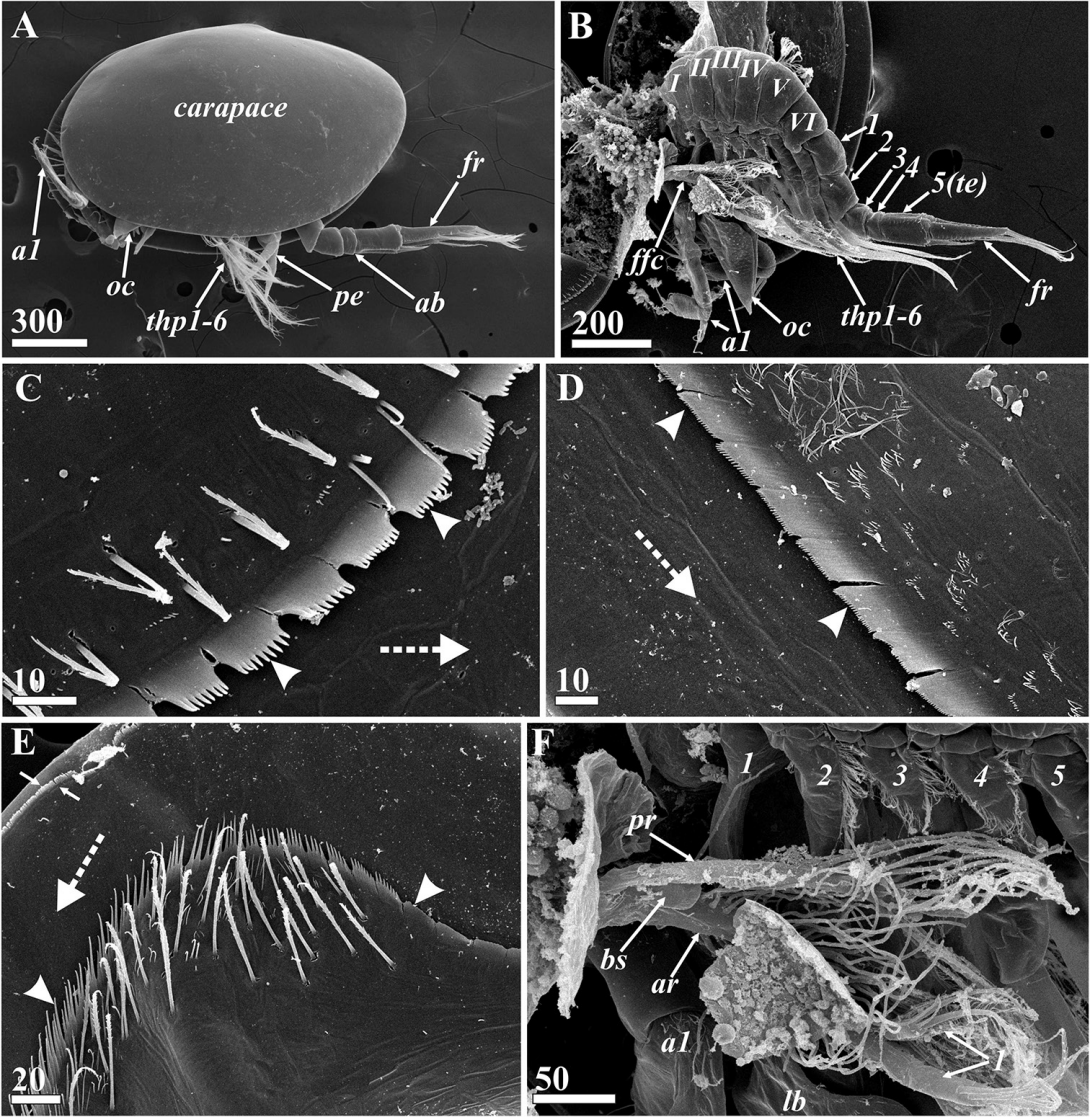
*Synagoga arabesque* sp. nov., male. General morphology, mantle structures (SEM) **A** general view lateral, left side **B** Inner body (prosoma), lateral view (thoracic segments numbered in Roman numerals, abdominal segments in Arabic numerals) **C** submarginal fold of mantle with cuticular fringe (indicated by arrowheads) near anterior margin (anterior direction indicated by dotted arrow) **D** submarginal fold of mantle with cuticular fringe (indicated by arrowheads) in middle part on ventral side of valve of carapace (anterior direction indicated by dotted arrow) **E** submarginal fold of mantle with cuticular fringe and setiform projections (indicated by arrowheads) at posterior end of valve of carapace (thin marginal fold indicated by small arrows, anterior direction indicated by dotted arrow) **F** frontal filament complex (thoracopods numbered). Abbreviations: *a1* – antennules, *ab* – abdomen, *ar* – anterior ramus of frontal filament complex, *bs* – basal ramus of frontal filament complex, *ffc* – frontal filament complex, *fr* – furcal rami, *lb* – labrum, *oc* – oral cone, *pe* – penis, *pr* – posterior ramus of frontal filament complex, *te* – telson, *thp1-6* – thoracopods I–VI. Scale bars: in μm.

**Figure 14.**
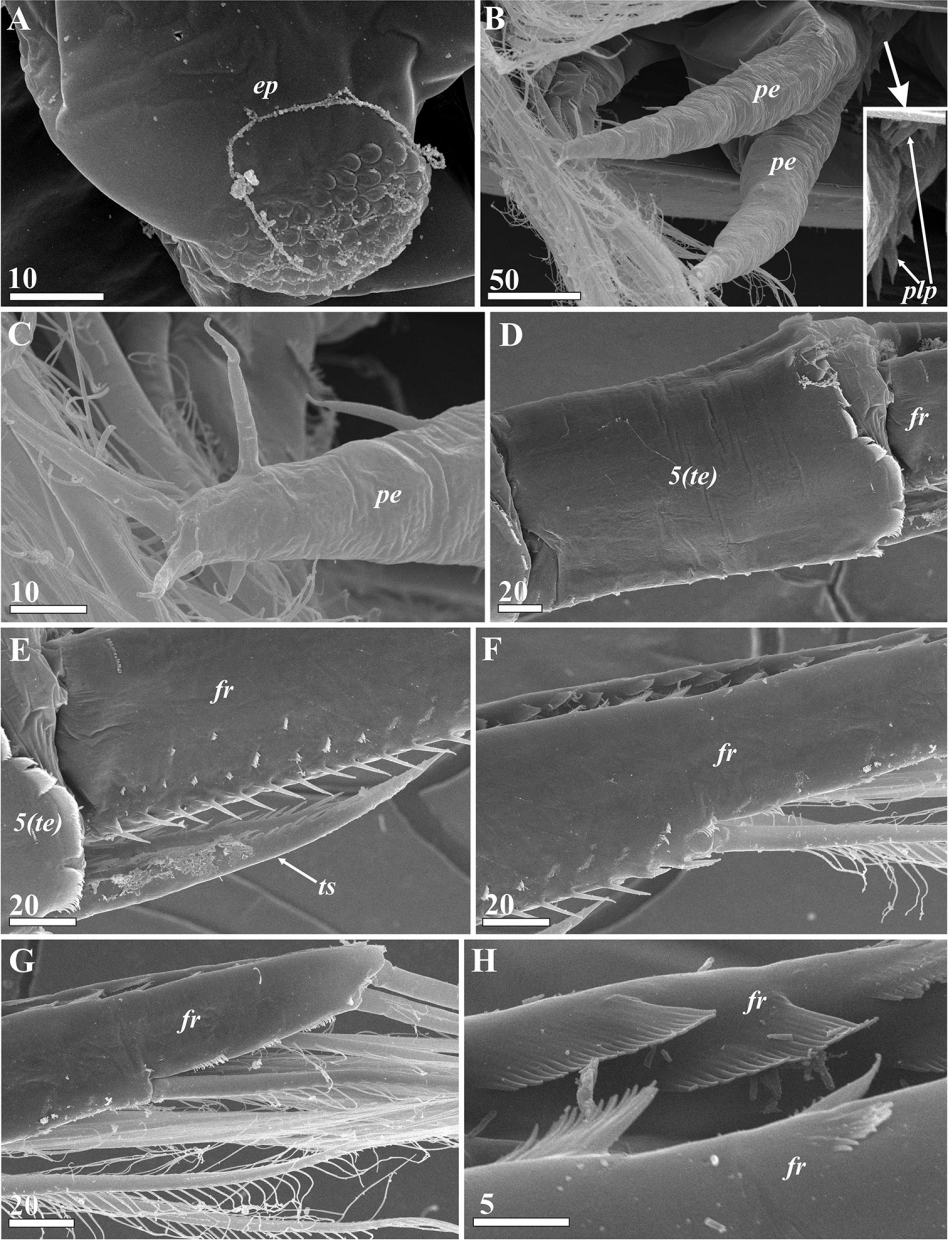
*Synagoga arabesque* sp. nov., male. Morphology of epaulet, penis, telson and furcal rami (SEM) **A** epaulet of thoracic segment 6 **B** rami of penis; enlarged terminal parts of pleural processes of first abdominal segment in rectangle area **C** tip of ramus of penis **D** telson, lateral side **E** telson spines and base of furcal ramus **F** middle parts of furcal rami **G** terminal parts of furcal rami **H** ctenoid scales on dorsal sides of furcal rami. Abbreviations: *ep* – epaulet, *fr* – furcal rami, *pe* – penis, *plp* – pleural process of first abdominal segment, *te* – telson, *ts* – telson spines. Scale bars: in μm.

**Figure 15.**
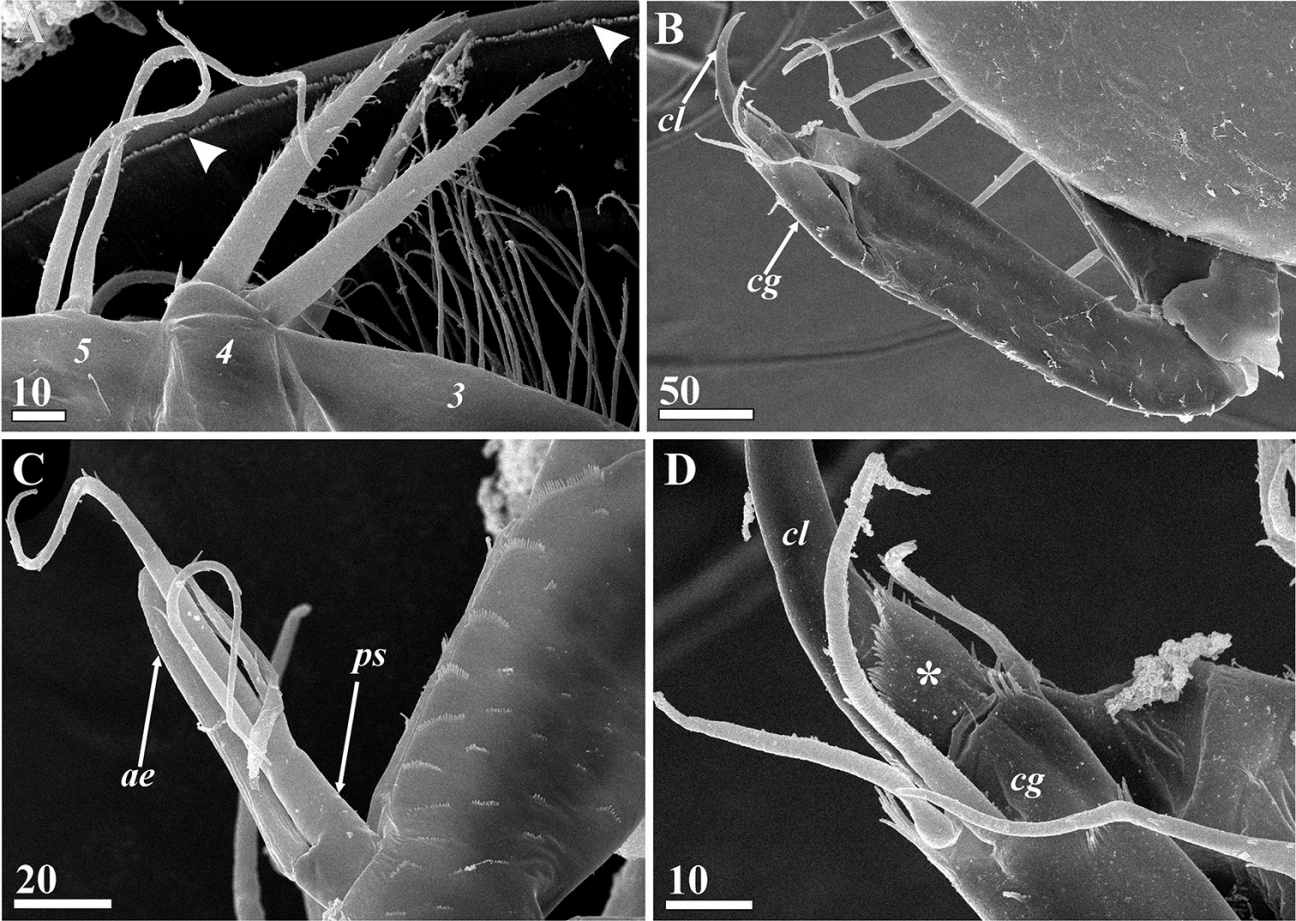
*Synagoga arabesque* sp. nov., male. Morphology of antennules (SEM) **A** spines of fourth segment forming ‘fork’ to accept claw of sixth segment (antennular segments numbered, marginal fold of mantle indicated by arrowheads) **B** sixth segment, left antennules, outer surface **C** proximal sensory process of sixth segment **D** junction between claw and claw guard showing their microsculpture, terminal ctenoid fold of claw guard sheathed claw indicated by asterisk, outer side. Abbreviations: *ae* – aesthetasc, *cg* – claw guard; *cl* – claw; *ps* – proximal sensory process. Scale bars: in μm.

**Figure 16.**
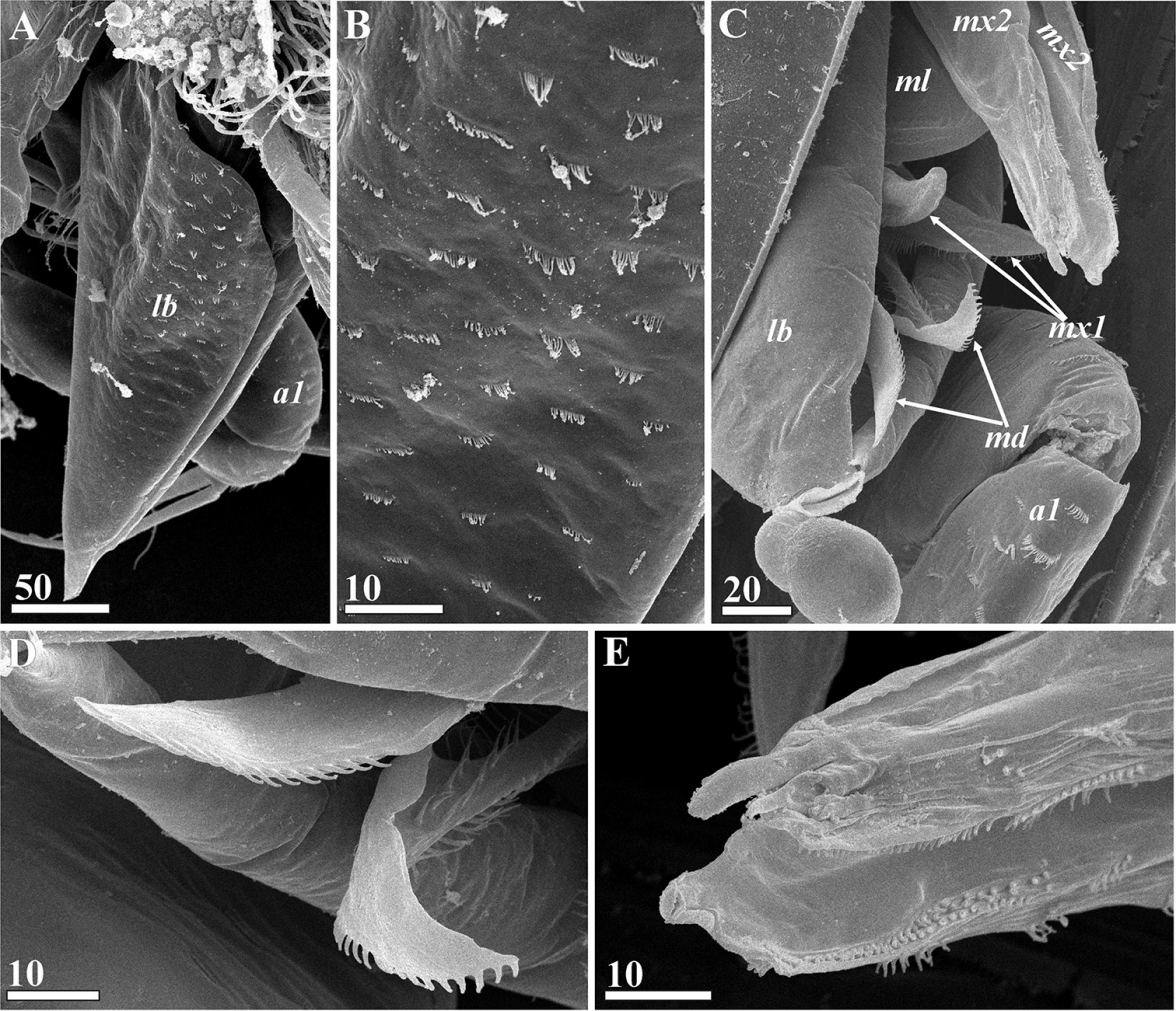
*Synagoga arabesque* sp. nov., male. Mouth parts (SEM) **A** labrum, posterio-lateral view, anterior margin left **B** lateral surface of labrum **C** distal part of oral cone with exposed tips of mouth parts **D** tips of mandibles **E** tips of maxillae. Abbreviations: *a1* – antennules, *lb* – labrum, *md* – mandible, *ml* – medial languette, *mx1* – maxillule, *mx2* – maxilla. Scale bars: in μm.

**Figure 17.**
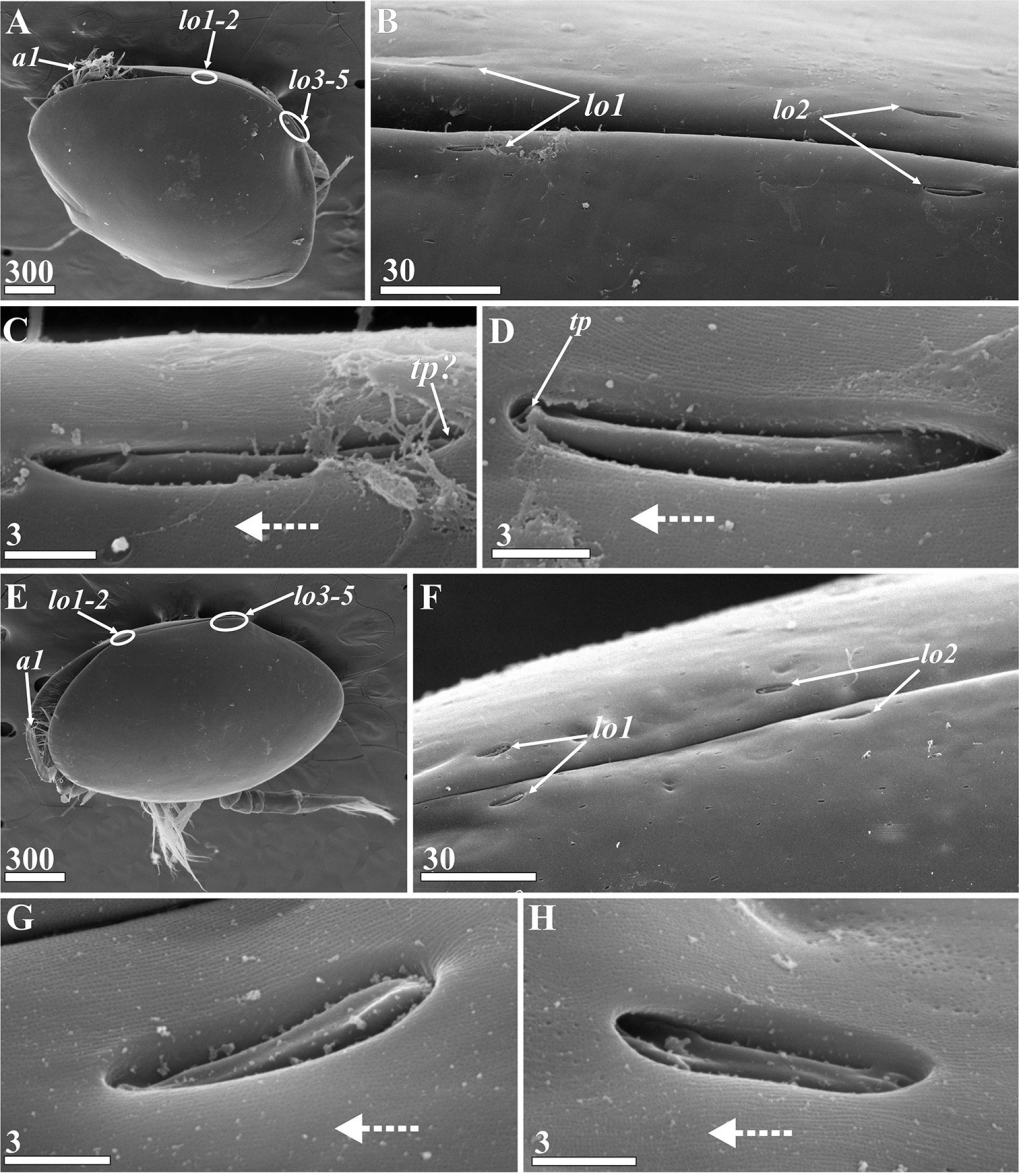
*Synagoga arabesque* sp. nov. Lattice organs, with dotted arrows indicating anterior direction (SEM) **A–D** female **E–H** male **A, E** general view, dorsolateral view, locations on carapace of anterior (1, 2) and posterior (3–5) pairs of lattice organs indicated by oval outlines **B, F** anterior lattice organs (1, 2) **C, G** left lattice organs 1 (first pair) **D, H** left and right lattice organs 2 (second pair). Abbreviations: *a1* – antennules, *lo1-5* – lattice organs, *tp* – terminal pore of lattice organ. Scale bars: in μm.

**Figure 18.**
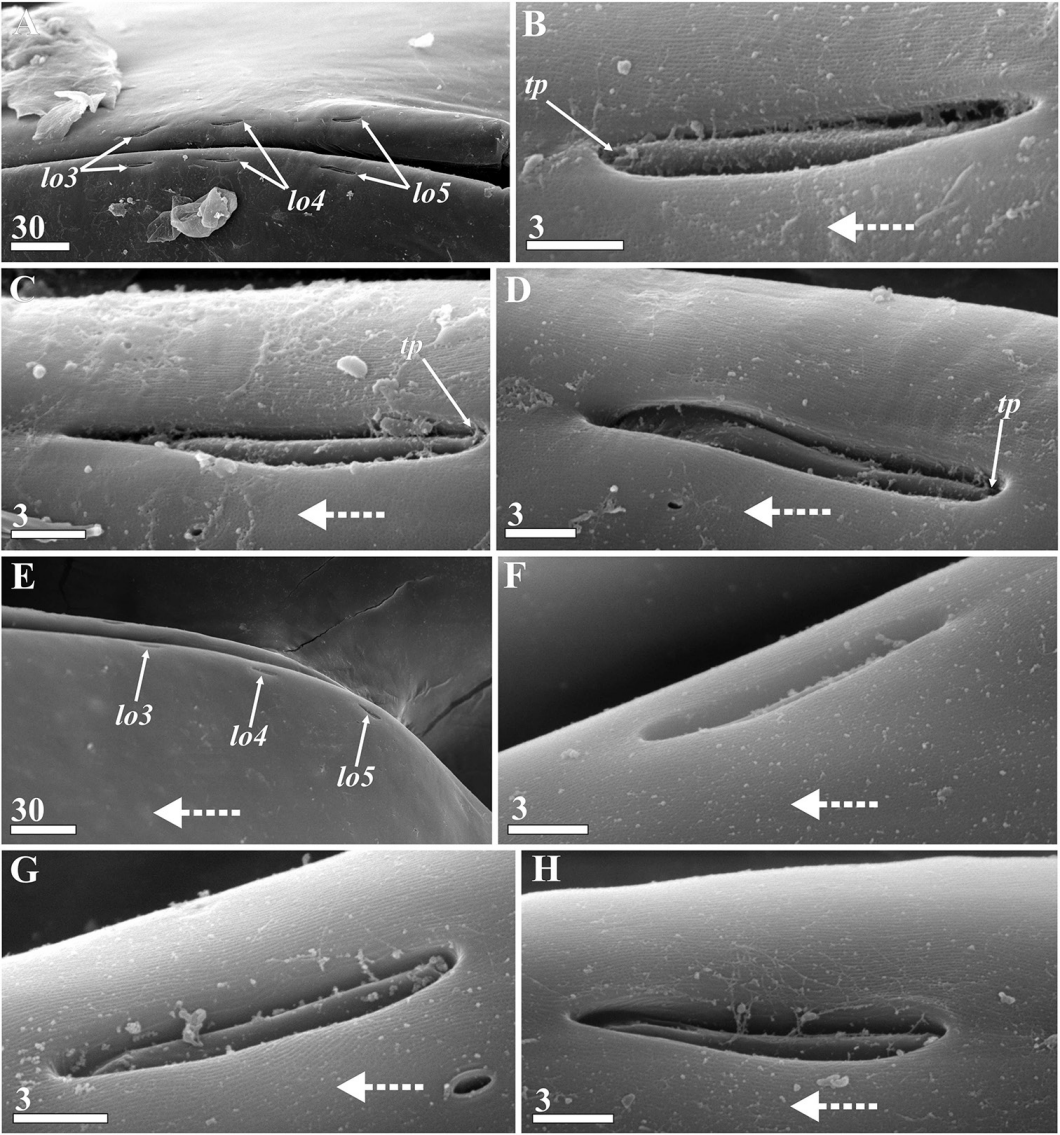
*Synagoga arabesque* sp. nov. Posterior (3–5) pairs of lattice organs, with dotted arrows indicating anterior direction (SEM) **A–D** female **E–H** male **A, E** posterior pairs (3–5) of lattice organs **B, F** left lattice organs 3 (third pair) **C, G** left lattice organs 4 (fourth pair) **D, H** left lattice organs 5 (fifth pair). Abbreviations: *lo3-5* lattice organs, *tp* – terminal pore of lattice organ. Scale bars: in μm.

Male (Figs 1D, 5–7, 13–16): Carapace bivalved, ellipsoidal, up to 1.5 mm long and 0.9 mm high, with slightly produced posterio-dorsal tip (Figs 5A, B, 13A). Dorsal margin almost straight; anterior, ventral and posterior margins rounded. Exterior of carapace smooth, lacking setae but covered with small pores (Figs 13A, 17E, F, 18E–H). Conspicuous deep pit with curved lumen opening on inner surface of anterior part of each valve (Fig. 5A, B). Gut diverticulum of simplified W-shape in comparison to female (Figs 1D, 5A, B), with 4 short lateral branches extending from anterior, posterior and ventral parts and terminated with light orange, widened areas at edge of carapace. Lobed testis within each carapace valve along lower part of gut diverticulum (Fig. 5A). Cuticular armament of mantle is similar to that of female (Fig. 13C–E). Edge of mantle forming thin marginal fold adjacent to margin of carapace and consisting of dense, tiny cuticular projections (Figs 13E, 15A). Anterior, ventral and posterior sides with submarginal underlying folder consisting of dense row of cuticular projections forming fringe or palisade, these projections longer in posterior side (Fig. 13C–E); anterior, ventro-posterior and posterior sides of mantle bearing setae with short setules (Fig. 13C, E).

Frontal filament long, trifid, more complex or less reduced than in female, with well-developed anterior and posterior rami covered by long, setiform cuticular projections (Figs 5C, 12B, F). Anterior ramus thicker and shorter than posterior, approximately 200–250 μm long; medial (basal) ramus short (50–80 μm), ampulliform, with smooth cuticle; posterior ramus longest, approximately 470 μm.

Body of male resembling that of female (Figs 5A, 13B): head bearing similar W-shaped antennules and well-developed oral cone; trunk consisting of 6 thoracic and 5 abdominal segments (Figs 5A, 13B, 14B); telson spines of same proportions and morphology (Figs 5F, 14E). Furcal rami resembling these of female in many details (Fig. 14E–H) but differ in having fewer long natatory setae on inner subdorsal margin (six instead of eight, Fig. 5F). Unlike in females, epaulets of sixth thoracic segment more strongly developed (Fig. 14A).

Condition of penis considerably different between male and female, tergite of penis-bearing first abdominal segment with conspicuous pair of long (approximately 100 μm), posteriorly directed pleural processes with four sharp terminal extensions that are absent in females (Figs 5D, E, 14B). Penis complex, approximately 600 μm long, ~ 4 times longer than supporting segment, and consisting of three parts: basal, medial and distal (Figs 5D, 14B, C). Basal shaft cylindrical, approximately 160 μm long. Medial part swollen, ~ 136 μm long, with unpaired thin process ~ 110 μm long extending from anterior side, tip of process (Fig. 5D) covered by thin layer of epicuticle. Distal part consisting of two rami originating from medial part and narrowing toward tips (Figs 5D, 14B, C). Cuticular setiform projections 10–20 μm long with apical pore (not setae) present along anterior margin of each ramus (Figs 5D, 14B, C). Tip of each ramus terminating in pair of these projections (Fig. 14C).

Antennules of male resembling those of female (Figs 6A, B, 15) but relatively thinner and longer with respect to body size. Second and third segments with dense, thin setae in same positions as in female. Two massive spines of fourth segment armed with row of conspicuous denticles along both anterior and posterior edges (Fig. 15A). Fifth segment with 4–6 rather than 6–9 setae on anterior margin (Figs 6A, B, 15B). Sensory and grasping structures of sixth segment of same morphology as in females, but ctenoid scales denser in lateral surfaces of segment (Figs 6B, 15C, D).

Oral cone and mouth parts similar to those of female (Figs 6C–G, 16), consisting of labrum (Figs 6G, 16A–C) enclosing an unpaired medial languette (Fig. 6D) and paired mouth parts, mandibles (Figs 6E, 16C, D), maxillules (Figs 6F, 16C) and maxillae (Figs 6G, 16C, E); tips of maxillules bifid, not harpoon-shaped, apical projection and adjacent process slightly larger than in females (Figs 6G, 16E). Thoracopodal setation of male (Table 1, Fig. 7) similar to that of female (Fig. 4) but showing some differences (only thoracopod VI have same setation); distal segment of exopod of thoracopod I with eight rather than seven setae (Fig. 7A), coxae of thoracopods II and III and bases of thoracopods II–IV have fewer setae along inner margins (Fig. 7B–D).

####### Lattice organs.

(Figs 17, 18): both sexes with five pairs of trough-like slits along hinge line of carapace (lattice organs: *lo1-5*, Figs 17, 18), situated co-linearly in two groups: anterior pairs 1–2 and posterior pairs 3–5. Those of both female and male are of similar morphology and arrangement and are therefore described together.

Lattice organs straight, each trough containing one short, modified seta (so-called crest) with terminal pore at free distal end (Figs 17D, 18B), terminal pore maybe hidden by debris, shrinkage or trough. Normally, each trough has oblique and rounded ends; distal part of crest lies at rounded end (Figs 17C, D, 18B–D). Cuticle of crests smooth, not perforated by small pores. Anterior lattice organs situated just posterior to point of divergence of carapace valves (Fig. 17A, E). *Lo1* 15 μm long in female (Fig. 17B, C) and 10–11 μm long in male (Fig. 17F, G), with posterior terminal pore, located 5–6 μm from hinge line (Fig. 17B, F). *Lo2* 100 μm behind first pair in female (Fig. 17B) and 80 μm behind in male (Fig. 17F), 16 μm long in female (Fig. 17D) and 10 μm long in male (Fig. 17H), with anterior terminal pore, located 9–10 μm from hinge line (Fig. 17B, D). Posterior lattice organs situated somewhat anterior to point of divergence of carapace valves, near their apices (Figs 17A, E, 18A, E), 530–550 μm behind anterior organs in mature female (Fig. 17A), 370–380 μm behind in male (Fig. 17E). *Lo3* 14–15 μm long in female (Fig. 18B) and 12 μm long in male (Fig. 18F), with anterior terminal pore, located 5–6 μm from hinge line. *Lo4* 25–28 μm behind *lo3* in female and 40–45 μm behind in male (Fig. 18A, E), 17–18 μm long in female (Fig. 18C) and 13–14 μm long in male (Fig. 18G), with posterior terminal pore, located 7–8 μm from hinge line (Fig. 18A). *Lo5* 45–50 μm behind *lo4* in female and 40 μm behind in male (Fig. 18A, E), 17 μm long in female (Fig. 18D) and 14 μm long in male (Fig. 18H), with posterior terminal pore, located 10–15 μm from hinge line.

####### Comparison.

Having both sexes of *S. arabesque* available makes it possible to compare this species with all other described species of *Synagoga*. The main characters used for comparison are given in Table 2. Owing to a lack of detailed description, no meaningful comparison with the juvenile “McKenzie’s larva” from the eastern Indian Ocean (cf. Grygier 1988) can be made. Only one species, *S. millipalus* represented by a single male, found in the Pacific Ocean off Okinawa, Japan. It differs in having fewer setae on the fifth antennular segment (three instead of four-six) and on the inner side of the furcal ramus (three instead of six), and also relatively longer telson spines (Grygier and Ohtsuka 1995). Only a single species, *S. normani* (based on a female), is known from the western Indian Ocean (Grygier 1983a). It has fewer setae on the fifth antennular segment (five instead of 6–9) and on the inner side of the furcal ramus (five or six instead of eight), and more setae on the second exopodal segment of thoracopod I (nine instead of seven). Four species inhabit the Atlantic and adjacent seas, these are *S. mira*, *S. bisetosa*, *S. paucisetosa* and *S. grygieri* (Norman 1888; Grygier 1983a, 1990a; Kolbasov and Newman 2018). The new species differs from *S. mira* (Norman 1888; Grygier 1983a) by having smooth, unscalloped edges of the gut diverticula, fewer setae on the fifth antennular segment (4–9 instead of 15), the second exopodal segment of thoracopod I (seven(eight) instead of 18) and the inner side of the furcal ramus (eight(six) instead of 14). It can be distinguished from *S. bisetosa* (Grygier 1990a) by having fewer setae on the fifth antennular segment (four-nine instead of ten), the second exopodal segment of thoracopod I (seven(eight) instead of ten) and the inner side of the furcal ramus (eight(six) instead of 13). The new species differs from *S. paucisetosa* (Grygier 1990a) in having more setae on the fifth antennular segment (four-nine instead of three) and the inner side of the furcal ramus (eight(six) instead of three); it also has relatively shorter telson spines. Finally, it can be distinguished from *S. grygieri* (Kolbasov and Newman 2018) by fewer setae on the fifth antennular segment of males (four to six instead of eight) and more setae on the inner side of the furcal ramus of females (eight instead of six); it also has relatively shorter fifth antennular segment and telson spines.

**Table 2.** Main diagnostic characters of species of the genus *Synagoga* (modified from Kolbasov and Newman 2018). The finding of *S. normani* on alcyonarian *Dendronephthya* is questioned, because all other congeners attributed to hosts were found on antipatharians.

Species characters	*S. mira* Norman, 1888	*S. normani* Grygier, 1983	*S.* sp. of Grygier 1988	*S. bisetosa* Grygier, 1990	*S. millipalus* Grygier & Ohtsuka, 1995	*S. paucisetosa* Grygier, 1990	*S. grygieri* Kolbasov & Newman, 2018	*S. arabesque* sp. nov.
Location, host, number and size of specimens	Naples, depth unknown, on *Parantipathes larix*, several males, 4×3 mm	Mombasa, 20 m, on *Dendronephthya* (?), single female, 1.73×1.32 mm	off West Australia, in plankton, host unknown, juvenile, 0.79×0.53 mm	outside Gibraltar, ca. 2000 m, host unknown, single immature female, probably male (has male penis), 2.8×2.2 mm	off Okinawa, between 575 m and surface, host unknown, single male, 1.7×1.25 mm	equatorial mid-Atlantic, 3459 m, host unknown, single male, 2.04×1.64 mm	Azores & Cape Verde Is, 20–40 m, on *Antipathella wollastoni*, males & females (app. 3+7), (1.8×1.25mm – male, 2.5×2.0 mm – female)	Green Island, off east Taiwan, 35 m, on *Myriopathes* sp., males & females (6+6), (1.5×0.9 mm – male, 2.3×2.0 mm – female)
shape of 4^th^ segment of a1	Subtriangular (anteriorly acuminate)	Triangular (posteriorly acuminate)	Rectangular	Rectangular	Triangular (posteriorly acuminate)	Triangular (posteriorly acuminate)	Rectangular in male, triangular (posteriorly acuminate) in female	Rectangular in male and female
armament at base of massive setae of 4^th^ segment of a1	Many very short spines	Very short, single spine	Absent	Absent	Single spine	Single spine	Both sexes with several very short spines – ctenoid scales	Both sexes with several very short spines – ctenoid scales
relative sizes of 5^th^ and 6^th^ segments of a1	approximately equal	5^th^ slightly shorter	5^th^ longer	approximately equal	5^th^ shorter	5^th^ shorter	ca. equal	5^th^ shorter
number of setae on anterior margin of 5^th^ segment of a1	15	5	5	10	3	3	9 (female)	6–9 (female)
							8 (male)	4–6 (male)
number of setae on 2^nd^exopodal segment of T1	18	9	11	10	5	5	7 (female)	7 (female)
							9 (male)	8 (male)
length of telson spine	Median length	Median length	Median length	Short	Very long	Very long	Median length	Short
number of medial setae on inner face of furcal ramus	~14	5 or 6	3	13	3	3	6	8 (female)
								6 (male)
gut diverticula	Low, frilly W-shape without major bifurcations	W-shaped with anterior and posterior arms bi- and tri-furcate respectively	None mentioned	Rounded W	Not observed	Rounded W	W-shaped with numerous branches in females. Males with less elaborate W-shape	W-shaped with numerous branches in females. Males with less elaborate W-shape
position of aesthetasc seta of proximal sensory process of a1	Subbasal?	Subbasal	Basal	Terminal	Basal	Subbasal	Subbasal	Subbasal
number and size of terminal setae on ramus of male penis	?	Inapplicable	Inapplicable	2 tiny	4 long	Few (3?) medium	2 medium	2 medium

## Discussion

Morphology of both sexes including external ultrastructure, as well as sexuality, host specificity, and biogeography of the genus *Synagoga* have been recently discussed in detail (Kolbasov and Newman 2018). In the present study we are providing new data on the structure of the lattice organs and anterior sensory pits of carapace, host specificity and biogeography of *Synagoga*. Developed anterior sensory pits (Figs 1A, 5A, B, 8E, F) are found on the inner side of valves in adult stages of both sexes of genera *Synagoga* and *Sessiligoga* (Grygier 1990b; Grygier and Ohtsuka1995; Kolbasov and Newman 2018; unpublished data). They are considered as possibly homologous to the pair of large anterio-ventral pores found externally on the ventral faces of the carapace valves of both sexes of both species of *Waginella*, *Waginella sandersi* (Newman 1974) and *Waginella metacrinicola* (Okada 1926), as well as two undescribed species of this genus (Newman 1974; Grygier 1990c; Itô and Grygier 1990; unpublished data). A chemosensory function was putatively proposed for these structures (Kolbasov and Newman 2018). Small pores and conspicuous volcano-shaped papillae observed on the surface of the canal of these pits in *S. arabesque* sp. nov. (Fig. 8E, F) may also be evidence in favor of chemosensory function.

In adults of both sexes of *S. grygieri* and *Synagoga arabesque* sp. nov. and the male of *S. millipalus*, all five pairs of lattice organs are situated co-linearly along the hinge line of the carapace valves, i.e., parallel to the hinge. A fully co-linear arrangement of the lattice organs has been considered plesiomorphic for ascothoracidans and also for all thecostracans (Jensen et al. 1994; Høeg and Kolbasov 2002; Celis et al. 2008; Kolbasov and Newman 2018). Apart from both *S. grygieri* and *S. millipalus* having the anterior terminal pore in *lo1* and posterior terminal pore in *lo2*, the new species has the posterior terminal pore in *lo1* and the anterior terminal pore in *lo2*. Thus, only posterior pairs of lattice organs (*lo3*, *lo4*, *lo5*) share the same position of terminal pores in the studied species of the genera *Synagoga* and *Sessilogoga* (Grygier and Ohtsuka 1995; Kolbasov and Newman 2018; herein; unpublished data). Species of both *Synagoga* and *Sessilogoga* share anterior terminal pores in *lo3* and posterior terminal pores in *lo4* and *lo5*. This is opposite to the condition in most thecostracans, which have a posterior terminal pore in *lo3* (e.g., Jensen et al. 1994; Kolbasov et al. 1999; Høeg et al. 2004; Celis et al. 2008), and thus represents a potential synapomorphy of these two genera (unpublished data). The different position of terminal pores of the lattice organs even within congeners (terminal pores of anterior lattice organs in *Synagoga*) shown here for the first time might be evidence that the configuration of lattice organs in ascothoracidans is not constant, at least in adult stages.

Four of the seven described species of *Synagoga* are attributed to particular hosts (Table 2) and three of them (*S. mira*, *S. grygieri*, and *Synagoga arabesque* sp. nov) were found on antipatharians. This may be evidence of the host specificity of *Synagoga* as exclusive ectoparasites or small predators of black corals. Therefore, we consider the attribution of *S. normani* to the alcyonarian host *Dendronephthya* as a possible misinterpretation. Grygier (1983a) described a single isolated female of *S. normani* ‘collected by P. Hutchence from alcyonacean coral, *Dendronephthya* sp.’ in Mombasa harbor and forwarded to him. We suspect that this record *Dendronephthya* may be of a non-specific substrate rather than an actual specific host for this species.

*Synagoga arabesque* sp. nov. is the second species of the genus found in the north part of the west Pacific after *S. millipallus*. Despite this fact, the new species resembles *S. grygieri* recently described from the Atlantic Ocean, Macaronesia (Kolbasov and Newman 2018; Table 2 herein) in many details. This may indicate that both *Synagoga arabesque* sp. nov. and *S. grygieri* evolved from a common ancestor and that the genus *Synagoga* exhibits the major Tethyan reliction pattern that is also characteristic of some ascothoracidans and barnacles (Newman and Ross 1971; Newman and Tomlinson 1974; Foster 1981; Kolbasov 2009; Kolbasov et al. 2015). Currently, studies of diversity of Ascothoracida are still based mainly on morphological approaches, future directions can involve molecular techniques to examine cryptic diversity and population genetics of Ascothoracida (see approaches in Chai et al. 2017; Chang et al. 2017; Ma et al. 2018; Jung et al. 2018)

## Supplementary Material

XML Treatment for Synagoga

XML Treatment for Synagoga arabesque
